# Dissecting seed pigmentation-associated genomic loci and genes by employing dual approaches of reference-based and k-mer-based GWAS with 438 *Glycine* accessions

**DOI:** 10.1371/journal.pone.0243085

**Published:** 2020-12-01

**Authors:** Jin-Hyun Kim, Joo-Seok Park, Chae-Young Lee, Min-Gyun Jeong, Jiu Liang Xu, Yongsoo Choi, Ho-Won Jung, Hong-Kyu Choi

**Affiliations:** 1 Department of Medical Bioscience, Dong-A University, Busan, Republic of Korea; 2 Department of Agricultural Biotechnology, National Institute of Agricultural Sciences, Rural Development Administration, Jeonju-si, Jeollabuk-do, Republic of Korea; 3 Department of Applied Bioscience, Dong-A University, Busan, Republic of Korea; 4 Systems Biotechnology Research Center, Korea Institute of Science and Technology (KIST), Gangneung, Republic of Korea; 5 Department of Molecular Genetics, Dong-A University, Busan, Republic of Korea; University of Guelph, CANADA

## Abstract

The soybean is agro-economically the most important among all cultivated legume crops, and its seed color is considered one of the most attractive factors in the selection-by-breeders. Thus, genome-wide identification of genes and loci associated with seed colors is critical for the precision breeding of crop soybeans. To dissect seed pigmentation-associated genomic loci and genes, we employed dual approaches by combining reference-based genome-wide association study (rbGWAS) and k-mer-based reference-free GWAS (rfGWAS) with 438 *Glycine* accessions. The dual analytical strategy allowed us to identify four major genomic loci (designated as *SP1*-*SP4* in this study) associated with the seed colors of soybeans. The k-mer analysis enabled us to find an important recombination event that occurred between subtilisin and I-cluster B in the soybean genome, which could describe a special structural feature of *i*^*i*^ allele within the *I* locus (*SP3*). Importantly, mapping analyses of both mRNAs and small RNAs allowed us to reveal that the subtilisin-CHS1/CHS3 chimeric transcripts generate and act as an initiator towards ‘mirtron (i.e., intron-harboring miRNA precursor)’-triggered silencing of chalcone synthase (CHS) genes. Consequently, the results led us to propose a working model of ‘mirtron-triggered gene silencing (MTGS)’ to elucidate a long-standing puzzle in the genome-wide CHS gene silencing mechanism. In summary, our study reports four major genomic loci, lists of key genes and genome-wide variations that are associated with seed pigmentation in soybeans. In addition, we propose that the MTGS mechanism plays a crucial role in the genome-wide silencing of CHS genes, thereby suggesting a clue to currently predominant soybean cultivars with the yellow seed coat. Finally, this study will provide a broad insight into the interactions and correlations among seed color-associated genes and loci within the context of anthocyanin biosynthetic pathways.

## Introduction

The soybean [*Glycine max* (L.) Merr.] is the most agro-economically important among all domesticated legume crops. The soybean has served as a main source of protein and oil for humans during its 4500 year-long history of domestication [1]. In addition to its nutritional value, the soybean contains a variety of bioactive phytochemicals such as saponin, lecithin, isoflavone, anthocyanin and many others [2]. Among other phytochemicals, anthocyanin is one of the key components that exert antioxidant effects and determine the seed colors of soybean. A recent study reported that dark-colored seeds (i.e., seeds with higher anthocyanin contents) had positive effects for the grain storage compared to light-colored seeds [3], because anthocyanins and other polyphenolic compounds contributed to resisting stresses during the storage [4,5]. As a result, longer seed storability leads to beneficial effects on seed germination, growth vigor, nutritional contents, and overall seed quality. Thus, the seed color should be considered one of the most critical qualitative traits for grain crops like soybeans. The seed coat of the soybean can be represented by four colors; black, brown, yellow, green, and additionally mottled. Among various cultivated soybeans, yellow soybeans are currently the most predominant. On the other hand, black soybeans are recently attracting more interest due to their antioxidant properties and flavors [6].

It is known that soybean seed color is controlled by multiple loci of moderate complexity. So far, at least nine genomic loci (*I*, *R*, *T*, *W1*, *O*, *D1*, *D2*, *qSC1* and *psbM*) have been reported [6–11], many of which are involved in anthocyanin-derived pigmentation pathways. For example, *I* locus harbors a cluster of chalcone synthase (CHS) genes, which are the key regulatory enzymes of the flavonoid pathway [12]. Four alleles (*I*, *i*^*i*^, *i*^*k*^, *i*) have been identified within the *I* locus, and they participate in controlling the colors of hilum and seed coat. Soybeans with the dominant *I* allele result in colorless (i.e., yellow or green) seed coats, whereas soybeans harboring the *i* allele exhibit colored (i.e., black or brown) coats. The *i*^*i*^ allele controls restricted pigmentation to the hilum, thereby resulting in soybean seeds with a colorless seed coat and colored hilum, while the *i*^*k*^ allele restricts pigments to the saddle region [13]. The *T* locus contains flavonoid 3’-hydroxylase (F3’H) and may control pubescence color. Soybeans with dominant *T* allele give rise to brown pubescence while those with the recessive *t* allele inherit gray pubescence [7]. A R2R3 MYB transcription factor (R2R3 MYB TF) was reported as a key player of the *R* locus that was associated with a brown seed coat and hilum [10]. *W1* locus is presumed to encode flavonoid-3’,5’ hydroxylase (F3’5’H). Under a *iRt* genetic background, dominant *W1* and recessive *w1* alleles are supposed to produce imperfect black and buff seed coats, respectively [11,14]. Both *D1* and *D2* loci, known as homologs of the *STAY-GREEN* (*SGR*), were revealed to be duplicated by recent whole genome duplication of the soybean and correlated with chlorophyll degradation [9,15]. Double-recessive mutant (*d1d1d2d2*) caused chlorophyll retention and exhibited the green seed coat color [9]. The *qSC1* locus is also known to be associated with a green seed coat color. However, genes associated with the corresponding phenotype have not yet been identified [6]. Another study reported that the *psbM* gene encoded one of the small subunits of photosystem II and that it was associated with the mechanism of chlorophyll degradation. Soybeans with the recessive allele of *psbM* gene are maternally inherited because this gene resides in the chloroplast [8].

Since the advent of next-generation sequencing (NGS) technology and production of the whole genome reference sequence of soybeans [16], many soybean cultivars, landraces, and wild types have been sequenced for the purpose of genome-wide analyses. Currently, a total of 3021 accessions derived from whole genome resequencing (WGR) are available in the public domain and account for a total data amount of 23.87 TB (the International Nucleotide Sequence Database Collaboration or INSDC: http://www. insdc.org/) [17]. Phenotypic data of the soybean can be obtained from other DB resources such as Germplasm Resources Information Network (GRIN: https://www.ars-grin.gov/) and RDA-Genebank Information Center (http://genebank.rda.go.kr/).

Genome-wide association study (GWAS) is a useful and powerful means by which we can identify genes and/or loci associated with traits of interest across the entirety of genomes. Conventionally, GWAS is performed using genomic variants such as single nucleotide polymorphisms (SNPs) and short insertion/deletion (InDels) obtained by mapping NGS reads against the reference genome. This type of ‘reference-based’ GWAS (rbGWAS) analysis fully relies on the completeness and correctness of the reference genome information. Due to this reason, rbGWAS may sometimes have limitations in applying the analytical tool for some species whose reference genomes are not available or are incomplete. Although rbGWAS has successfully served to discover numerous trait-associated variants [18–20] in the past, considerable cases of ‘phenotype-to-genotype parallelism’ could not be explained due to a problem known as ‘missing heritability’ [21].

To effectively overcome the limitation in rbGWAS methodology, a new approach, namely ‘reference-free GWAS or rfGWAS’ that does not require the reference genome, has recently been developed [22–27]. This analytical approach employs all possible sub-nucleotides, called ‘k-mers’, with optimized lengths, all of which can be identified within the whole genome NGS reads. Depending on the presence or absence of k-mers, researchers can identify variants and determine their types. It appears that the majority of rfGWAS approaches have been applied to bacterial genomes [22–25], while being rarely applied to human genomes [26]. Recently, plant genomes have been analyzed with these methods [27].

In this study, we intended to employ dual approaches, in terms of analytical means and data combinations, to accomplish precision analysis in genome-wide detection of genes and/or loci associated with the pigmentation of soybean seeds. Towards this direction, k-mer-based rfGWAS in the soybean genome was employed to complement rbGWAS and to ensure discovery of missing trait-associated genomic fractions, by involving a wide array of NGS data combined with in-house-generated and public data of WGR, RNA-seq and small RNAs.

## Materials and methods

### Plant materials, DNA extraction and NGS sequencing

A total of 43 Korean soybean accessions, including 40 cultivars and 3 landraces (seed coat colors; 22 black, 2 brown, 16 yellow and 3 green) were chosen for a new WGR data production. All of the seed materials were acquired from the Rural Development Administration (RDA)-Genebank Information Center (GBIC; http://www.genebank.go.kr) in Korea. Detailed information on each accession is available in S1 Table. Sterilized seeds were sown in 1 L pots and grown under greenhouse conditions. Leaves at the R1~R2 growth stages (i.e., during the flower development) were sampled for DNA extraction. Genomic DNA was extracted using the Plant DNA isolation kit (GeneAll, Seoul, ROK) according to the manufacturer’s protocol. Purity and integrity of DNA were inspected by A260/A280 absorbance ratio and agarose gel electrophoresis, respectively. The sequencing libraries of each accession, with an average insert size of 550 bp, were constructed according to the manufacturer’s instruction (Illumina, San Diego, CA, USA). Using the Illumina HiSeq4000 NGS platform, paired-end reads were produced at the Macrogen Inc. (http://www.macrogen.com, Seoul, Korea).

### Collection of NGS data and phenotype information

To avoid false positive variants generated by incorrect mapping due to highly duplicated soybean genome (approximately 75%) [28], only paired-end reads were collected from the European Nucleotide Archive (ENA, http://www.ebi.ac.uk/ena) and the National Agricultural Biotechnology Information Center (NABIC, http://nabic.rda.go.kr/) with following search options; library strategy = WGS, depth ≥ 5.0, read length ≥ 90bp. As a result, 662 and 121 WGR data sets were obtained from ENA and NABIC, respectively. Phenotype data was retrieved mainly from GRIN (https://www.ars-grin.gov/), RDA-GBIC (https://www.ars-grin.gov/) DB and related literatures as well. To identify alternative splicing by nucleotide variations, RNA-seq paired end reads were collected from ENA with following parameter adjustment; library strategy = RNA-Seq, depth ≥ 5.0, read length ≥ 90bp.

### WGR read mapping, variant calling and annotation

Trimmomatic (http://www.usadellab.org/cms/?page=trimmomatic, Version: 0.36) was used to remove low quality regions of raw reads with the following parameter options (Leading:3 Trailing:3 SlidingWindow:4:5 Minlen:90) according to the previous report [29]. *G*. *max* Williams 82 assembly v2.0 was downloaded from the Phytozome (https://phytozome.jgi.doe.gov/pz/portal.html#!info?alias=Org_Gmax), which contained only nuclear genome information, and was used as the reference genome for mapping. For other sub-organelle genome information, data for mitochondrion (https://www.ncbi.nlm.nih.gov/nuccore/NC_020455.1) and chloroplast (https://www.ncbi.nlm.nih.gov/nuccore/NC_007942.1) genomes were separately downloaded from the NCBI. These nuclear and sub-organellar genome information were merged together and used for the whole genome backbone for mapping WGR reads. This way, we intended to avoid any possibility of erroneous mapping that might be caused by non-nuclear DNA. Paired-end reads were mapped to the backbone reference genome using Burrows Wheeler Aligner (BWA) program (http://bio-bwa.sourceforge.net/, Version: 0.7.15-r1140) with default options [30]. Overall mapping rates were calculated by Samtools software (http://samtools.sourceforge.net/, Version: 1.3.1). Samples with <80% mapping rates were removed according to previously reported guideline [31]. Picard software (https://broadinstitute.github.io/picard/, Version: 2.8.3) was used to sort mapping outcomes and to remove duplicated reads.

Reads mapped around InDel sites were realigned using the IndelRealigner program, and scores for base-pair quality were recalibrated using the BaseRecalibrator program, both of which were available as package programs in Genome Analysis Toolkit (GATK, https://software.broadinstitute.org/gatk/, Version: 3.7-0-gcfedb67) [32]. Variant discovery was conducted using HaplotypeCaller in the GATK package. To minimize false positive variant calling, preliminary raw variants were further filtered out with following parameter options; QD < 5, FS ≥ 30, SOR ≥ 3.0, MQ ≤ 30, MQRankSum < -3.5, MQRankSum > 3.5, ReadPosRankSum < -2.0 and ReadPosRankSum > 2.0. For the purposes of annotating genes and evaluating effects of variant, SnpEff (http://snpeff.sourceforge.net/, Version: 4.3t) was employed [33].

### Population and phylogenetic analysis

In order to interpret population structure of 438 soybean accessions, a total of 5717575 SNPs were used and calculated with analysis options of MAF>0.05 and 10% maximum missing data. Initially, a neighbor-joining tree was constructed using Molecular Evolutionary Genetics Analysis (MEGA, https://www.megasoftware.net/, Version: 7.0.26). A consensus tree with 1000 bootstrap replicates was plotted for high-precision evolutionary relationship analysis [34]. To infer the population structure, fastSTRUCTURE (https://rajanil.github.io/fastStructure/, Version: 1.0) program was used and analyzed using the variational Bayesian framework. The fastSTRUCTURE program was implemented initially with simple basic option for K values ranging from 1 to 10. The ‘chooseK.py’ script was used to determine optimal number of genetic components, followed by ‘distruct.py’ script to visualize admixture proportions [35].

### Reference-based GWAS analysis

For the purposes of precisely searching for trait-associated variants, two sets of NGS-derived data were employed for the GWAS analyses; 1) WGR data from 438 soybean accessions, 2) Illumina Infinium SoySNP50K BeadChip SNP data [36] derived from 20087 soybean accessions. The SoySNP50K SNP data were downloaded directly from the SoyBase (http://soybase.org/snps). To remove low quality and less informative variants, variants with MAF<0.05 and 10% minimum missing data were filtered out. Data filtration was able to identify a total of 6948411 and 36587 nucleotide variants from the WGR and array chip data sets, respectively. GWAS analysis was conducted using the Efficient Mixed-Model Association eXpedited program (EMMAX, http://genetics.cs.ucla.edu/emmax/, Version: 20120210) [37]. The kinship matrix was measured by EMMAX-IBS. In order to implement GWAS, EMMAX program requires a binary input of data; for example, 1) quantification of contrasting qualitative phenotypes, 2) classification of phenotypic characters into either ‘case’ or ‘control’. In case of traits related to more than two phenotypes, EMMAX may give rise to a problem for the GWAS analysis. To circumvent such a problem, all possible binary combinations (‘case’ or ‘control’) of phenotypes were made and used for the GWAS analyses. To estimate the significance threshold of GWAS results, the Bonferroni correction method was employed.

### 3-D structure molecular modeling of the CaaXEP protein

3-D molecular structures of the CaaXEP protein were predicted using the stand-alone package of I-TASSER server with default options [38]. In order for the molecular modeling, protein sequences were fed into the program after removing the chloroplast transit peptide sequences. Among five predicted models suggested by the I-TASSER, the best structure was selected on the basis of the number of decoy for robust ranking factor. The resulting data in the pdb file format were visualized using the PyMOL program [39]. The secondary structures (i.e., helix, sheet and loop) were drawn by the ‘cartoon and surface’ mode within the overall structural integrity of corresponding 3-D structure.

### K-mer-based reference-free association analysis

To conduct reference-free association analysis, K-mer Counter (KMC) software (http://sun.aei.polsl.pl/kmc/, Version: 3.0) was used to count all possible k-mers in all WGR reads with following parameter options;-k31, -m256 [40]. K-mers counted only once was filtered out to obtain accurate data set, because they might contain sequencing errors. Resulting k-mers were combined and converted into variant call format (VCF) data using in-house program (available at github.com/kimzz14/Kmer-to-VCF). Subsequently, GWAS analysis was performed using PLINK program with following parameter options;—allow-extra-chr,—allow-no-sex—assoc (http://zzz.bwh.harvard.edu/plink/, Version: 1.90b5.2) [41].

### mRNA-seq mapping analysis

To investigate the effects of nucleotide variants on structural changes in genes, mRNA-seq reads were mapped onto the backbone reference genome using the STAR program (https://github.com/alexdobin/STAR, Version: 2.7.0d) [42]. To figure out alternatively spliced gene structures of interest, RNA-seq reads were assembled into transcripts using StringTie program (https://ccb.jhu.edu/software/stringtie/, Version: 1.3.3b) with parameter ‘-G’ [43]. Finally, aligned reads and assembled transcripts were visualized using Integrative Genomics Viewer (IGV; https://software.broadinstitute.org/software/igv/, Version: 2.4.7 [44]. The Sashimi plot was generated by IGV with a parameter of minimum junction coverage 5.

### Small RNA-seq mapping analysis

Because adapter sequences used for generation of small RNA-seq reads were heterogeneous and were not available in public domains, they were searched by k-mer-based method and subsequently removed from the small RNA-seq reads using in-house program. The sizes of trimmed small RNA-seq reads ranged from 14 to 43 nucleotides with a major peak around 21~24 nucleotides in length. Trimmed small RNA-seq reads were mapped onto the backbone reference using STAR program with the option of ‘no mismatches allowed’ [42]. Subsequently, the python language was used for visualizing alignment results [45].

### Isolation and quantification of anthocyanins

Soybean seeds were ground and sieved through a 55 mesh screen. 40 mg of seed powder was mixed with 70% aqueous ethanol (4mL, 1% HCl) containing 1.5 μg/ml phlorizin as an internal standard, sonicated for 30-min, and set at room temperature in the dark for 16 hours. 2 mL supernatant was filtered through a 0.20-μm syringe filter with the GH Polypro membrane (Pall Corporation, Ann Arbor, MI, USA), freeze-dried under the vacuum, and re-dissolved using 0.5 mL of 50% aqueous ethanol for the LC/MS analysis.

Anthocyanins were quantified using the MicrOTOF-Q II (Bruker Daltonics, Bremen, Germany) mass spectrometer coupled with the 1290 HPLC system (Agilent Technologies, Santa Clara, CA, USA). Resolving power of the MS instrument was 17500 at m/z 922. The compositions of solvent system were as follows; solvent A- water/acetonitrile (95:5, v/v with 0.1% formic acid), solvent B-acetonitrile/water (95:5, v/v with 0.1% formic acid). A 45-min linear gradient was applied; initially 10 to 60% increment of solvent B for 35 min, followed by a steeper increment of 60 to 100% solvent B for 10 min. Chromatographic separation was performed using reverse phase Aegispak C18 Column (250×4.6mm, 5-μm) at flow rate of 0.7 ml/min.10-μL aliquot of each extract was injected into the mass spectrometer, and spectra were monitored ranging from 150~1000 m/z with 1.0 sec interval under the positive ESI mode. The ion source parameters for high-resolution mass measurements were as follows; +4.5 kV for the capillary voltage, 10 L/min of the nebulization gas flow, 180°C as a source gas temperature and 35 V for the cone voltage. A calibration curve for each anthocyanin was plotted by calculating, based on the weighted least squares regression analysis, the ratio of peak areas between analyte/internal standard and the nominal concentration of analyte in 50% blank ethanol.

## Results

### Genetic and phenotypic diversity of 438 *Glycine* accessions

**Fig 1:**
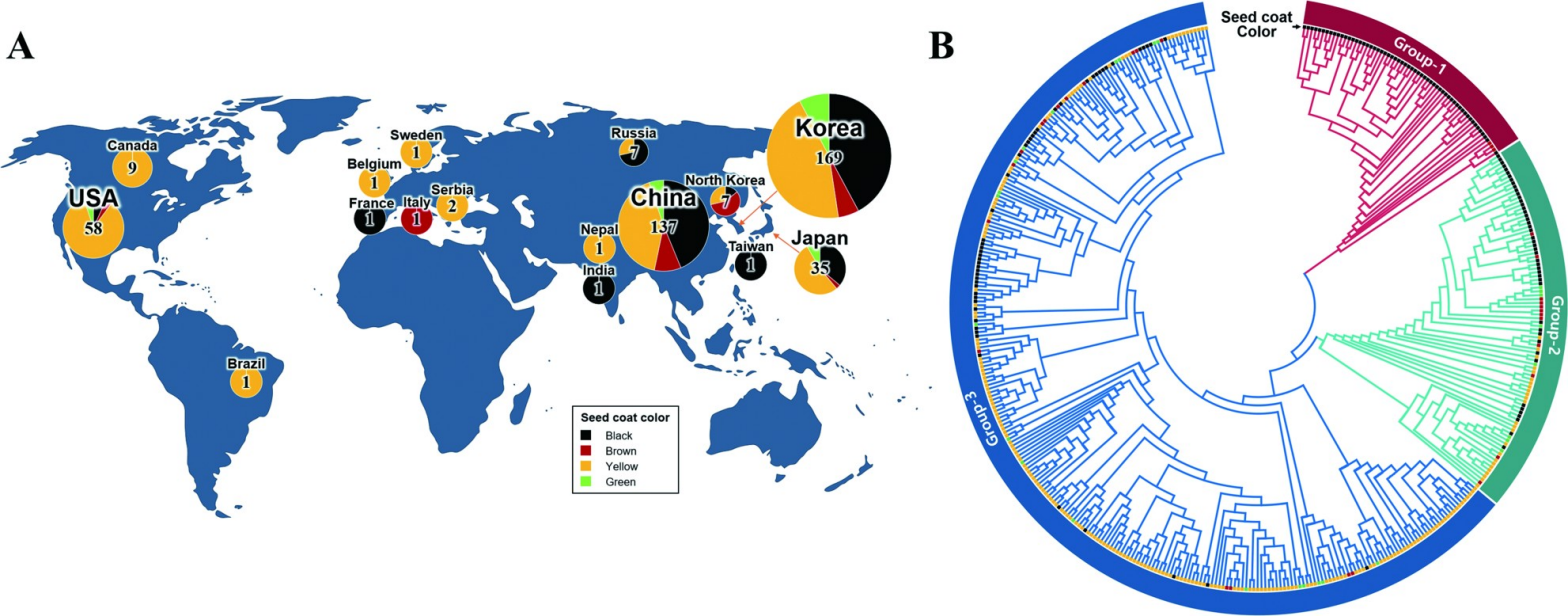
Geographic, genetic and phenotypic diversity of 438 soybean accessions. (A) Geographic and phenotypic distribution of 438 soybean cultivars. (B) Phylogenetic relationships among 438 soybean accessions. The seed color of each soybean accession is depicted in the second circle.

Originally, we acquired WGR data from a total of 783 *Glycine* accessions (662 plus 121 accessions from ENA and NABIC, respectively) available on the public databases(DB). Additionally, WGR data for 43 soybean accessions were newly generated in this study (NCBI SRA accession ID; SRR767070-SRR7637112) and combined with the public data, thereby accounting for a total of 826 WGR data sets. Of these, 739 accessions (89.5%) were correctly mapped to the soybean reference genome (Williams 82 assembly v2.0), while phenotype information could be obtained only for 438 accessions (59.3%) via public DBs and literatures (S1 Table). According to geographical origins, the reorganized WGR data consisted of 169 Korean accessions, 137 Chinese accessions, and 132 accessions from other countries (Fig 1A). Resulting 6.7 Tb sequence data, with an average depth of 15.7 x for each genome, were analyzed using the GATK standard protocol. This analysis allowed us to identify a total of 5717575 SNPs and 961113 InDels (S2 Table).

The phylogenetic topology of neighbor-joining tree demonstrated that 438 *Glycine* accessions (*G*. *max*, 381 accessions; *G*. *soja*, 57 accessions) could be grouped into three main clades, which were mainly correlated with their evolutionary relationships rather than geographical locations (Fig 1B). Consistent with this phylogenetic investigation, the fastSTRUCTURE analysis also resulted in an estimated value of K = 3 as the optimal number of subpopulations (S1 Fig). Within the whole context of phylogeny, the majority of wild soybeans (i.e., *G*. *soja*, 96.5% [55/57]) were clustered into Group-1, almost all of which had black seed coat with tawny pubescence. Group-2 was mainly composed of landraces with various seed coat colors, while the majority of them had black seed coat. Different from other groups, Group-3 consisted of cultivars and landraces with various seed colors, while the majority of those exhibited the yellow seed coat. It was notable that the frequency of black seed color decreased gradually from Group-1 to Group-3, whereas the occurrence of yellow seeds increased in an opposite manner. It was also observed that yellow hilum and gray pubescence were increasingly selected during the development of soybean cultivars from the wild species. Taken together, both population structure and phylogenetic investigation indicate that seed pigmentation-associated trait was appreciably influenced by selection on seed colors during the history of domestication processes in soybean cultivars (Fig 1, S3 Table).

### Reference-based GWAS analysis on soybean pigmentation-associated traits

**Fig 2:**
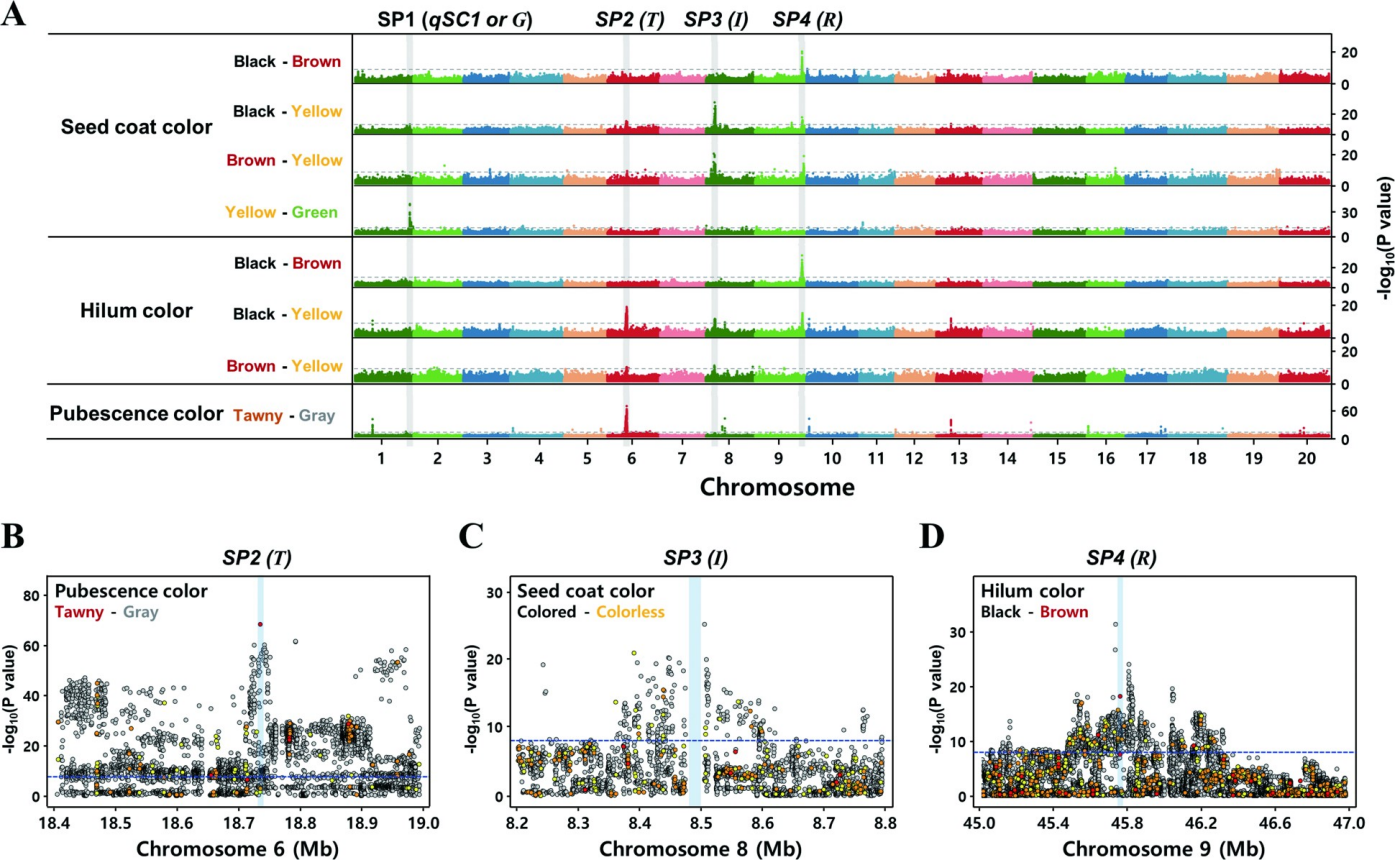
GWAS analysis on the seed coat, hilum and pubescence color. (A) Genome-wide Manhattan plots of the GWAS analyses. The statistical significance of P values is depicted by the negative logarithm with the threshold (gray dotted lines) of 5 x 10^−9^. Magnified Manhattan plots of candidate genomic region at the T locus (B), I locus (C) and R locus (D). Each colored circle denotes a different level of the SnpEff-defined (a simple putative impact assessment) functional impact of individual variants: red, high; orange, moderate; yellow, low; gray, modifier.

For the purposes of in-depth identification and cross-confirmation of causative variants associated with soybean seed pigmentation, two different sets of data were employed for the GWAS analysis. Both data sets contained 6678688 variants (>6.6M SNP data) from 438 WGR data and 36587 variants (>36K SNP data) for the SoySNP50K array data derived from 20087 soybean accessions, respectively. Each data set showed different frequencies and distributions of nucleotide variations throughout the genome. SNPs of 438 WGR data were distributed relatively more randomly or evenly, with an average frequency of SNP/158bp, compared to that of the SoySNP50K array data (S2 Fig). In contrast, it was shown that SNP distribution of the SoySNP50K array chip corresponded mainly on gene-rich genomic regions, presumably due to intentional variant selection around proximal regions of genes (S2 Fig). For precise prediction of trait-associated genomic loci through GWAS statistical analysis, reasonably large numbers of samples and variants are essential. Thus, we intended to complement GWAS analysis with one another by employing two different variant call data sets (i.e., 438 WGR data with higher number of SNPs and SoySNP50K array chip data with higher number of soybean accessions). Towards this direction, statistically significant signals were filtered and cross-checked using both data sets, and shared portions of GWAS analyses were extracted to cross-check the results. In order for qualitative traits like seed pigmentation to be analyzed based on the linear mixed model (LMM), quantified binomial terms, such as ‘case’ or ‘control’, for the qualitative traits are required. However, LMM-based GWAS analysis is not suitable for simultaneously dissecting qualitative traits represented by three or more phenotypes. To dissolve such a limit, we created all possible combinations that could be distinguished by the ‘case/control’ binomial quantitative characters for three contrasting phenotypes (i.e., colors for the seed coat, hilum, and pubescence), and performed the GWAS analyses for each combination with corresponding data (Fig 2, S3 Fig). As a result, four seed pigmentation (SP)-associated loci (designated as *SP1*-*SP4* in this study) were identified in four chromosomes (i.e., chromosomes 1, 6, 8 and 9). In addition, genomic loci for three other qualitative traits, including pod color, flower color, and growth habit, were identified (S4 Fig).

Detailed information on seed color-associated GWAS results is summarized in Table 1 and S4 Table. Of these four loci, *SP2* locus was revealed to be involved in the pubescence color and to span a genomic range of 17683675~19248187bp in Chromosome 6 (Table 1). Within this region, the most statistically significant variant, which could affect the function of the protein product, was found at 18737366 bp. This nucleotide alteration resulted in a frameshift caused by a single base deletion (i.e., TC→T) on F3’H gene, which was previously known as *T* locus (Table 1). Consistent with the previous report, the single base deletion altered its reading frame and introduced a premature termination, consequently resulting in a truncated F3’H protein in which lost GGEK consensus sequence and heme-binding domain essential for its function [7]. Occurrences of the truncated protein products were different depending on their pubescence colors; 1.55% (4/258) and 84.62% (143/169) among soybeans with tawny and gray pubescence, respectively (S4B Table). In addition, a new deletion variant (i.e., CA→C at Chr6_18737556 bp) was discovered in the same gene, which was positioned at 190 bp upstream of above-mentioned variant allele (S4B Table). This allele was also predicted to produce a cleaved F3’H protein. Overall frequencies of loss-of-function caused by these two truncated variants were 1.55% (6/258) and 88.76 (150/169) depending on tawny and gray pubescence colors, respectively (S4B Table).

**Table 1:** Information for key genes with the highest significance of variations at four seed color-associated loci.

Locus Name	Conven-tional Locus Name	Chr.	Genomic range			The most significant variants with high impact on protein functions						
			Start	End	Size (kbp)	Position	P-value	Allele 1	Allele 2	SnpEff Effect^a^	SnpEff Impact^b^	Gene ID & annotation
SP1	qSC1 or G	1	53,087,195	53,269,307	182	53,229,579	4.66E-39	G	A	Splicing site, structural change	High	Glyma.01G198500 (CAAX endopeptidase)
SP2	T	6	17,683,675	19,248,187	1,565	18,737,366	5.39E-20	TC	T	Frame shift	High	Glyma.06G202300 (flavonoid 3'-hydroxylase)
SP3	I	8	8,360,663	8,622,831	262	8,507,079	1.33E-25	G	A	Upstream	Modifier	Glyma.08G110500 (Chalcone synthase 4)
SP4	R	9	45,576,015	45,939,643	364	45,759,137	1.32E-12	TC	T	Frame shift	High	Glyma.09G235100 (R2R3 MYB transcription factor)

^a^Predicted functional effects by variant at the identified genomic site.

^b^Putative functional impact of variant that is defined by SnpEff

GWAS analyses identified the *SP4* locus near the terminal region of chromosome 9 and revealed that the locus was associated with brown pigmentation in both seed coat and hilum (Fig 2). Among many variant alleles found within this locus, a nucleotide variation with the highest probability and functional impact on protein synthesis was identified at 45759137 bp (Table 1, S4D Table). Further analysis could predict that the variant allele (a single base deletion of TC→T, and subsequent introduction of premature stop codon) would produce a truncated transcript of R2R3 MYB TF (Glyma.09G235100) (Table 1, S4D Table). It is known that the R2R3 MYB TF is a key member of previously reported *T* locus and regulates expression of UF3GT gene, which plays a critical role in the final step of anthocyanin biosynthesis [10]. It was also previously reported that UF3GT expression was repressed when the R2R3 MYB TF was altered by four different variants [10]. Consistent with Gillman’s data (10), we could detect the same three of four variants within the genic region of R2R3 MYB TF as follows: G→C at Chr9_45758856 bp, nonsynonymous mutation; CG→C at Chr9_45759100 bp, frameshift; GT→TT at Chr9_45759165, loss of splicing donor site (S4D Table). Consistent with this analysis, 98.2% (159/162) soybean accessions with normal R2R3 MYB TF exhibited black seed coats, while 100% (28/28) soybean accessions with altered genotypes resulted in brown ones. With regard to hilum phenotypes, soybean accessions also exhibited different frequencies of hilum colors; 91.90% (193/210) of black hilum and 98.61% (71/72) of brown ones without or with altered alleles in R2R3 MYB TF, respectively (S4D Table).

GWAS analysis with a reorganized population of yellow vs green seed coats allowed us to identify the *SP1* locus at the terminal region of chromosome 1, which was potentially associated with the phenotype of green seed coat (Fig 2, S5A Fig). Identification of *SP1* locus was cross-confirmed using the SoySNP50K array chip (S5A Fig). It was acquainted that *SP1* locus corresponded to *qSC1* locus that was one of 14 *qSC* loci previously mapped by QTL analysis using a bi-parental population [6]. It was known that dominant *qSC1* allele was involved in the phenotype of green seed coat, whereas recessive *qsc1* allele resulted in yellow ones. However, causative genic components have yet not been articulated probably due to a vast range of the *qSC1* genomic region (1079 Kbp) and relatively low statistical significance [6]. Nevertheless, we could considerably narrow down corresponding genomic region for the *qSC1* locus (182 Kbp) and identified six functionally significant candidate genes within this region (S4A Table, S5 Table). Among many other variants and 6 candidate genes, it was expected that a nucleotide alteration found at the RNA splicing site (i.e., AG→AA at Chr1_53229579 bp) of Glyma.01G198500 (annotated as CaaX-type endopeptidase or CaaXEP) should be the most functionally significant (S5 Table). To verify production of alternatively spliced transcript, RNA-seq data was collected from the public domains and mapped on the CaaXEP gene using StringTie program (https://ccb.jhu.edu/software/stringtie/) [43]. This transcriptome analysis allowed us to reveal that the alternative splicing site was generated 168 bp behind the original location and led to the creation of a new protein product exhibiting a different open reading frame and 3D-structure (S5B and S5C Fig). In this analysis, we independently discovered and articulated the CaaXEP gene (Glyma.01G198500) as a key player associated with the green seed coat. However, a recent study has already reported that the same gene (i.e., CaaXEP) was involved in the phenotype of the green seed coat and seed dormancy as well, which was denoted as ‘*G*’ locus [46]. This indicates that our study was efficient and accurate in detecting genomic loci and characterizing key genes associated with the seed pigmentation.

### In-depth GWAS analysis on *i*^*i*^ locus

For purposes of better interpreting the effect of the *I* locus on seed pigmentation with no perturbation by other loci, the population was reorganized for an optimal GWAS analysis. According to the phenotype distribution with regard to *T* locus, almost all soybean accessions (143/144 accessions, 99.31%) possessing truncated variant (*t* allele) of F3’H exhibited the colorless phenotype (i.e., yellow or green) in seed coat, thereby allowing us to assume that the truncated F3’H was intimately associated with colorlessness in seed coats (S6B Table). On the other hand, it is known that the *I* locus involves four different alleles (namely, *I*, *i*^*i*^, *i*^*k*^ and *i*), each of which exerts different effects on colors of seed coat and hilum [7,11]. Taken combined genetic properties together between these two loci, the population was sub-divided according to their phenotypes (i.e., seed coat, hilum, and pubescence) (S6 Fig), with an expectation that GWAS analysis with the re-organized population according to customized genotypic combinations could lead us to more accurate investigation on the *i*^*i*^ allele. Towards this direction, populations with the following phenotypic contrasts were used for rbGWAS analysis; ‘colored seed coat/colored hilum/brown pubescence’ vs ‘colorless seed coat/colored hilum/brown pubescence’. This customized-by-case rbGWAS led us to the identification of the *SP3* locus (conventionally *I* locus), which was mapped to 8360~8622 Kbp region of chromosome 8 (Fig 1, Table 1), and revealed that the corresponding genomic region was clustered with multiple copies of CHS genes. As widely known, CHS catalyzes the initial step of the phenylpropanoid pathway leading to the flavonoid biosynthesis and plays a pivotal role in regulating spatial/temporal production of anthocyanins and proanthocyanidins [7,11]. It was previously reported that the expression of CHS gene in soybeans with colorless seed coat was hampered by the posttranscriptional gene silencing (PTGS), consequently leading to the abolishment of chalcone production, which was the first stage precursor towards the synthesis of flavonoids and other secondary metabolites [13]. Among alleles found in this genomic region, a variant allele with the highest functional impact was detected in CHS4 (G→A at Chr8_8507079; Glyma.08G110500) (Table 1). However, this nucleotide variation could not account for the expected mechanism of the PTGS. Most perplexingly, a large unsequenced genomic segment, probably due to confounded read assembly by inverted repeat arrangement of multiple CHS genes, was noticed and hampered further analysis into searching for key trait-associated elements within the locus.

### Discovering a genomic structural change at *I* locus by k-mer analysis

Since rbGWAS could not further dissect detailed genomic structure of the *i*^*i*^ locus due to incomplete assembly around corresponding genomic region, k-mer-based variant calling method, which could effectively operate in reference-independent manner, was applied to scrutinize the genomic structures and to identify variations. As a prelude to verify reliability of variant data obtained from the k-mer-based analysis, all k-mers with optimal length consisting of 31 nucleotides were compared with variants derived from the reference-based variant calling method. The data comparison exhibited a reliable consistency between two different, but complementary, analytical methods (i.e., reference-based vs reference-free variant calling methods); for examples, SP1-99.54%, SP2-97.92% and SP4-96.58% (S6 Table).

To search for the missing genomic part of *i*^*i*^ locus, association analysis was performed using only variant data set, which was obtained from the k-mer-based variant calling. Of all k-mers, a unique k-mer with the consensus sequence of ‘ATGTCAATAAGATAAGTATAT TGTAAAATGG’ (named k-mer^*i*^) showed significantly different distribution between two above mentioned populations; 1.3% (2/154 accessions) in the population with phenotypes of ‘colored seed coat/colored hilum/brown pubescence’ vs 95.5% (64/67 accessions) in the population consisting of ‘colored seed coat/colorless hilum/brown pubescence’. To locate corresponding genomic position of the k-mer^*i*^, BLAST was carried out against two reference genomes; *G*. *max* Williams 82 (https://phytozome.jgi.doe.gov/pz/portal.html#!info?alias=Org_Gmax) and *G*. *soja* PI483463 (https://soybase.org/data/public/Glycine_soja/PI483463.gnm1.YJWS). However, the genomic position of k-mer^*i*^ could not be found in neither *G*. *max* nor *G*. *soja* genomes, implicating that one of two genomes was incompletely assembled around the k-mer^*i*^ site. Subsequently, BLAST was conducted against the non-redundant nucleotide database, in which contained the largest collection of nucleotide sequences. Such an attempt allowed us to find two *G*. *max* bacterial artificial chromosome (BAC) clones (i.e., BAC77G7-a and Gm_ISb001_104_J07).

**Fig 3:**
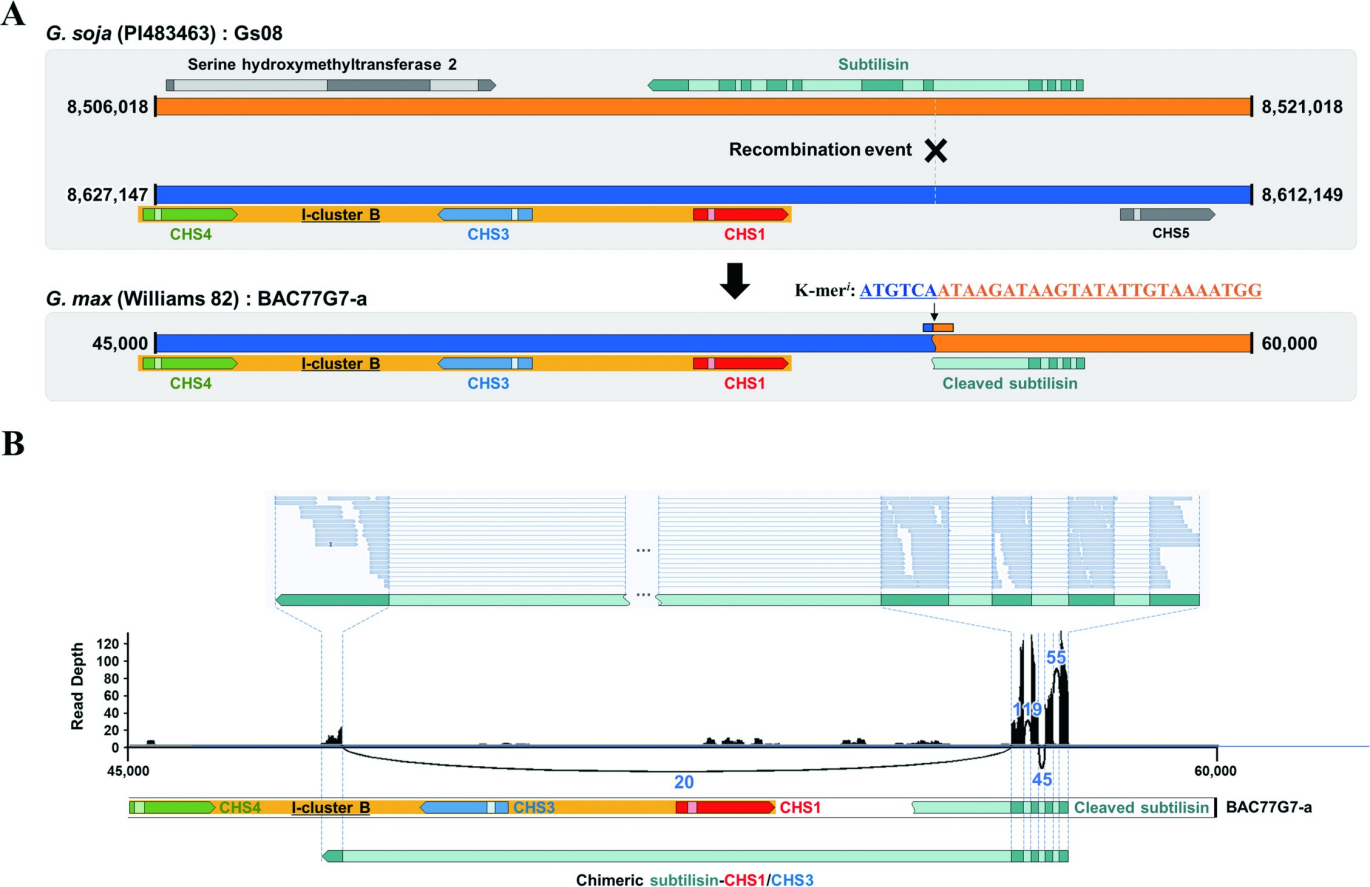
Genomic structures and gene expression profiles around the k-mer^i^ allele. (A) Comparative genomic structures between *G*. *soja* (PI483463) and *G*. *max* (Williams 82 BAC77G7-a) at the I-cluster B. (B) Gene expression profile and Sashimi plot of chimeric subtilisin-CHS1/CHS3 gene. X-axis indicates relative genomic locations within the BAC77G7-a BAC clone, while Y-axis the read depth. Solid lines are used to depict exon/intron junction-to-junction, and corresponding number of reads that are splitted due to the junctions are denoted on each line. The upper part of Sashimi plot demonstrates mapping result of mRNA reads with an emphasis on distribution of junction-spanning reads along on the chimeric subtilisin-CHS1/CHS3. The dark and light colors in arrow-shaped boxes represent the exons and introns of the gene, respectively.

It was found that BAC77G7-a clone was previously sequenced and analyzed to study the effects of CHS genes at *I* locus on the seed pigmentation [12]. Corresponding study found two inverted repeat clusters (namely, I-clusters A & B), each of which was arranged in the order of CHS1-CHS3-CHS4 vs CHS4-CHS3-CHS1, respectively. It was revealed that the k-mer^*i*^ newly identified in this study was localized on the right side of I-cluster B, which was the missing genomic part in the reference genome and thereby could not be properly analyzed by the rbGWAS approach (Fig 3A). In an attempt to find a causal genomic change, it was also revealed, by comparing I-cluster of BAC77G7-a with corresponding genomic region of *G*. *soja* (PI483463), that a critical recombination event had occurred at the *I* locus in *G*. *soja* genome, leading to the creation of a new recombinant allele, subsequent inheritance into *G*. *max* genome (Fig 3A) and thereafter selection for soybean cultivars with yellow seed coats. Coincidently, such a scenario of the new allele creation could be evidenced by the k-mer^*i*^ that positioned precisely onto the recombination genomic site (Fig 3A). Also, Cho et al (2019) confirmed that a recombination event occurred at the same location [47].

### Identifying a chimeric subtilisin-CHS1/CHS3 gene

It was noteworthy that the 5’-portion of truncated subtilisin gene was connected next to the I-cluster B due to the recombination event (Fig 3A). This resulted in a truncated subtilisin with a loss of 3’-portion of the gene, while retaining its 5’-portion and its own cis-elements, which might control the expression of rearranged chimeric gene. To verify a creation of the unexpected chimeric transcript, mRNA sequence data (SRR1174225; generated from the seed coat of *G*. *max* Williams 82) were obtained and mapped onto the BAC77G7-a BAC clone sequence. As a result, RNA-seq reads were mapped not only to the coding regions of truncated subtilisin but also, interestingly, to an unexpected genomic region located between CHS3 and CHS4, which was predicted to be a newly acquired exon (Fig 3B). In addition, we could identify a significant number of junction reads spanning the fourth exon of the truncated subtilisin and the newly found fifth exon positioned between CHS3 and CHS4 (Fig 3B). This result clearly demonstrates the creation of a new transcript reading frame, within which two CHS genes occupies the middle portion of newly acquired intron of the chimeric gene along with the gain of a new terminal exon (Fig 3B). Consequently, the RNA-seq mapping analysis provided a strong evidence that the recombination occurred at the *I* locus had eventually led to the creation of a new chimeric transcript enclosing both CHS1 and CHS3 genes (named as chimeric subtilisin-CHS1/CHS3 or SC1C3 gene). In addition, the sequence similarity between CHS1 and CHS3 including their introns is really high (>99%), and subsequently two very similar sequences, when aligned in an inverted orientation, can allow formation of a hairpin structure that may potentially cause the RNA interference [48].

### Discovering intron-containing primary miRNA via mirtron

**Fig 4:**
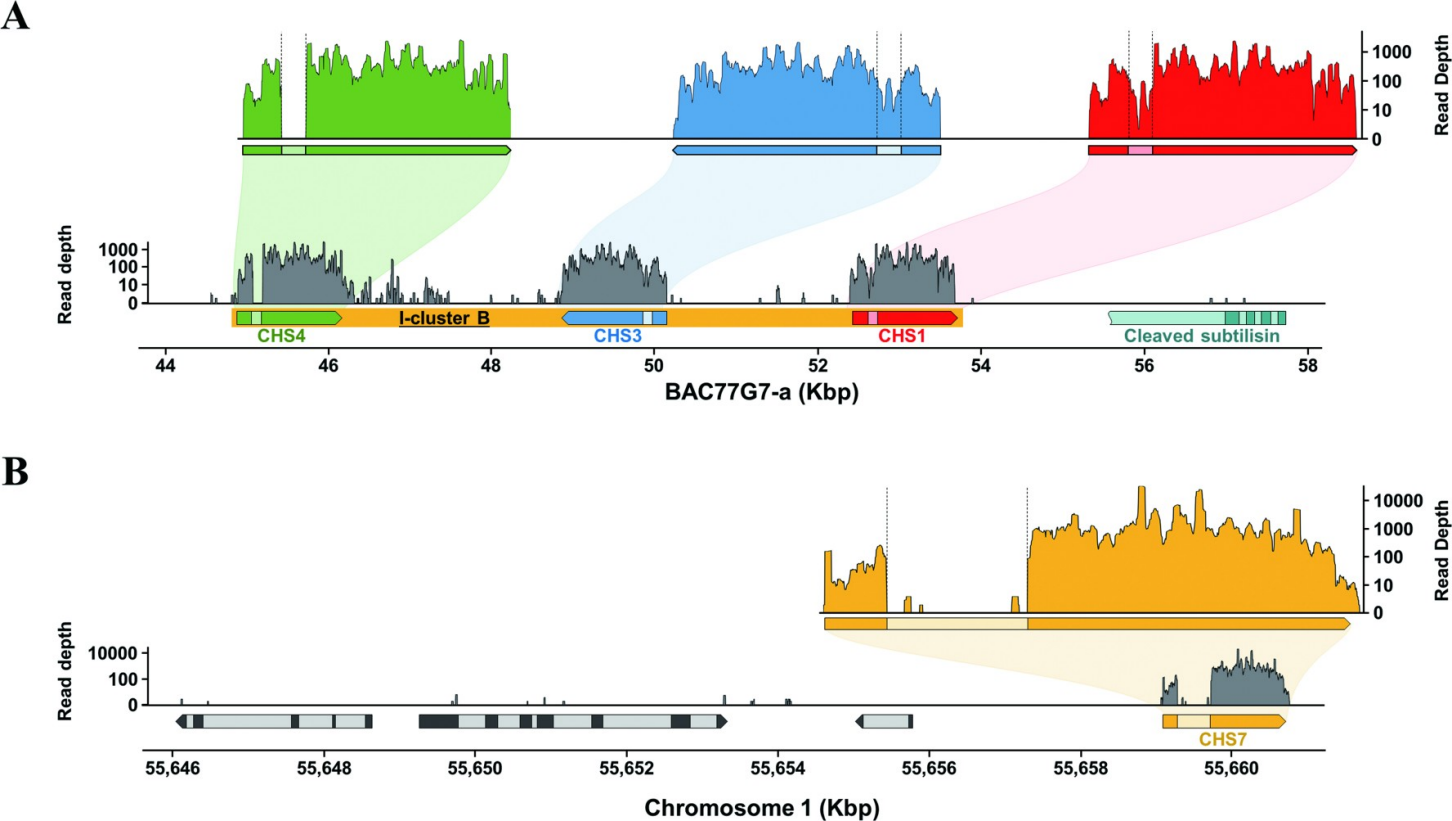
Mapping analysis of small RNA-seq reads on the I-cluster B (A) and CHS7 (B). Read depth was counted by the number of reads on each position. Exon-intron junctions are denoted with the vertical dotted lines. The size and position of genes are all drawn to scale, while the read depth to the logarithmic scale.

To confirm whether the chimeric SC1C3 gene is a key player responsible for the PTGS mechanism of CHS genes, NGS-derived small RNAs were further analyzed. Towards this direction, small RNA sequence data (SRR646505; generated from the seed coat of *G*. *max* Williams 82) were obtained and mapped onto both soybean reference genome and BAC77G7-a. As shown in Fig 4, the analysis focused on the distribution patterns across CHS genes, in which the majority of small RNAs were mapped along on the exon regions of CHS genes with high frequencies. In this analysis, it was most notable that a considerable portion of small RNA reads were mapped on the intron regions of CHS1 and CHS3, but not on introns of CHS4 and any other CHS genes (Fig 4, S7 Fig). This result strongly indicates that only two CHS genes (i.e., CHS1 and CHS3), which changed into a new intron of the chimeric SC1C3 gene, can play a certain role in the small RNA biogenesis via ‘mirtron (i.e., intron-containing miRNA precursor)’ formation. Naturally due to high sequence similarity and inverted orientation between CHS1 and CHS3, this finding allowed us to conceive a subsequent generation of mirtron-derived primary microRNAs (miRNAs) after the splicing of pre-mRNA derived from the chimeric SC1C3 gene.

In addition, it was expected that the primary miRNAs might stimulate the amplification of small RNA species in cells by inducing secondary small interfering RNAs (siRNAs) derived from mRNAs of all other CHS genes. As a supportive example, CHS-wide small RNA mapping analysis provides evidence that CHS7 transcript (also including other CHS genes) can be silenced by the mirtron-mediated secondary siRNAs. In more detail, CHS7 (chromosome 1) is totally separated from the *i*^*i*^ allele (chromosome 8) and has quite low sequence similarity with CHS1, where the sequence alignment of >20 bp long small RNAs exhibited only 7.43% (120/1615 bp of CHS7) of perfect matches between CHS1 and CHS7. Nevertheless, it was shown that a large portion of the small RNA reads was mapped to CHS7 (and/or CHS8 that has high sequence similarity to CHS7), but with no small RNAs mapped within its intron region (Fig 4B, S7 Fig). This result indicates that the small RNAs identified within CHS7 are all generated through miRNA-directed mRNA cleavage of mature CHS mRNAs, which is known as ‘phased small interfering RNA’ (phasiRNA) [49]. Cho et al (2013) suggested that secondary CHS siRNAs from the CHS7 target mRNA was amplified by primary CHS siRNAs which was generated from the inverted repeat cluster of chalcone synthase genes CHS1-3-4 [50]. However, our results show that secondary siRNA is amplified by primary CHS miRNA, not primary CHS siRNA.

## Discussion

Since the advent of high-throughput NGS sequencing technologies and large scale genotyping array platforms, GWAS has played a pivotal role in identifying informative trait-associated genes and genomic loci in many crops [6,18,51–54]. On the other side, many programs relevant to GWAS analysis, such as PLINK, EMMAX, GAPIT and MLMM, have been developed towards extracting more accurate genomic information within the context of phenotype-to-genotype correlations [37,41,55,56]. Nevertheless, the rbGWAS analyses can commonly be affected by the accuracy and completeness of reference genomes. In addition, the rbGWAS may sometimes mislead us to erroneous conclusions, if the reference genomes are misassembled due to their complex nature. Moreover, even if a certain genome is perfectly assembled as a standard framework for the reference, rbGWAS may retain their innate limitations against calling every single variation, mainly due to the genome’s complexity and large scale genomic changes unaccounted in common GWAS analyses (e.g., copy number variations [or CNV], inversions and translocations), all of which may cause the ‘missing heritability’ problem [21]. Therefore, it seems reliable that the k-mer-based rfGWAS may offer a new avenue or a means that can effectively complement the rbGWAS. The k-mer-based approach may exert undeniable advantages over common rbGWAS analyses by enabling us to discover any types of variants, including large scale structural variations and CNVs as well as common SNPs and InDels, regardless of the integrity of reference genomes. To our best knowledge, this study would be one of rare cases in which the k-mer-based association analysis was applied to discover trait-associated genomic variations in plants. By employing dual analytical approaches and phenotype-defined populations, four genomic loci (i.e., *SP1*~*SP4* loci) associated with the seed colors could be identified (Fig 2, S3 Fig). Of these four loci, it was proved that three loci (i.e., *SP1*, *SP2* and *SP4*) corresponded to the previously reported genomic loci (i.e., *qSC1/G*, *T* and *R* loci, respectively) (Table 1) [7,10,46]. Further in-depth analysis proved that the corresponding variant alleles were identified in key trait-associated genes at each locus.

**Fig 5:**
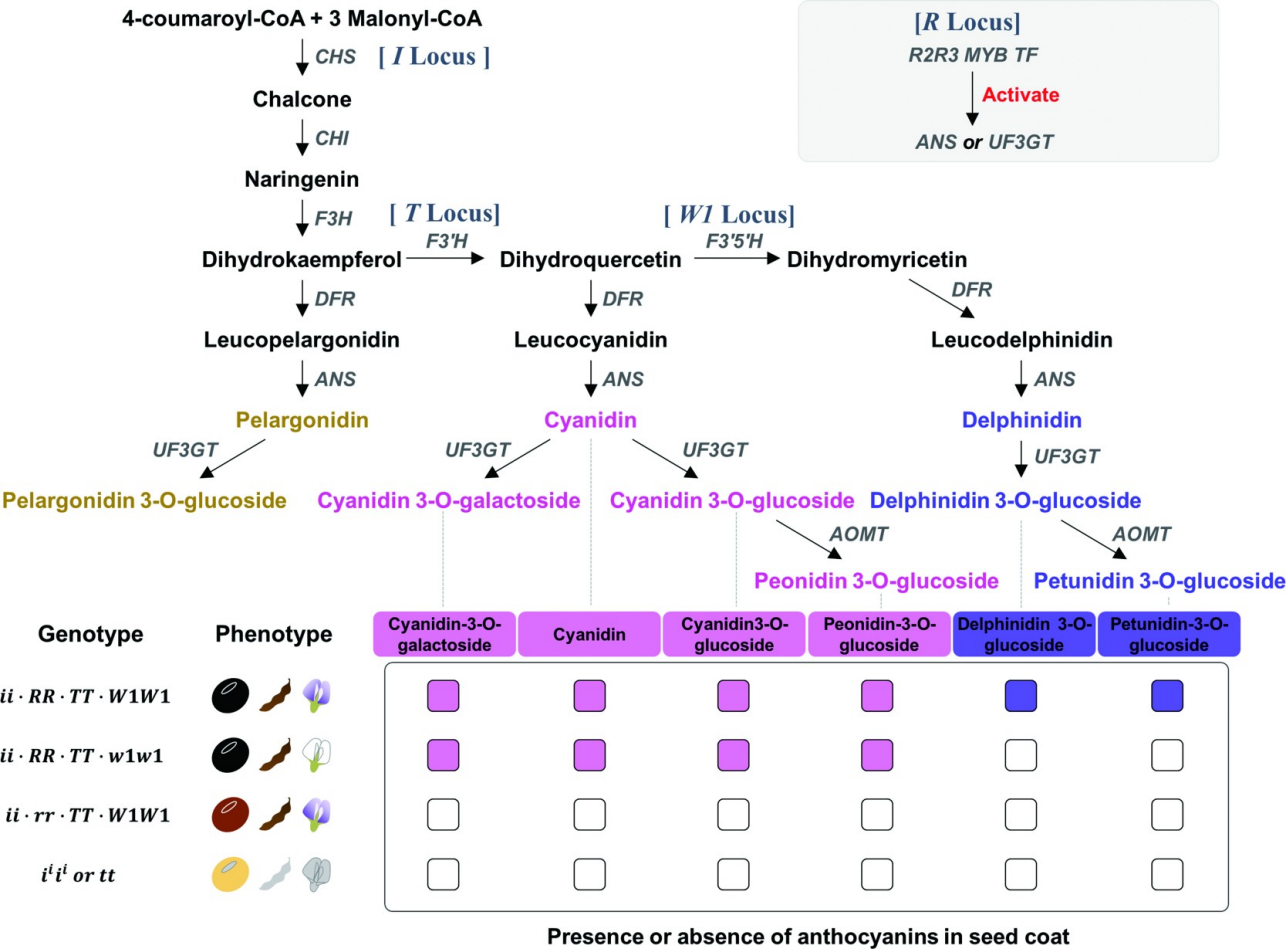
Schematic for the seed color-associated loci/genes within the context of the anthocyanin biosynthetic pathway. Genes or corresponding enzymes are denoted with the capital letters of abbreviated names, as follows: CHS, chalcone synthase; F3’H, flavonoid 3’-hydroxylase; F3’5’H, flavonoid-3’,5’ hydroxylase; CHI, chalcone isomerase; DFR, dihydroflavonol-4-reductase; ANS, anthocyanidin synthase; UF3GT, flavonoid 3-O-glucosyltransferase; AOMT, anthocyanin O-methyltransferase.

To get a more integrated picture of correlations and/or interactions among these genes and loci, a biochemical characterization was performed by measuring the contents of anthocyanins in 43 soybean accessions that were used for the WGR data production (S7 Table). Interactions among four seed color-associated loci within the context of metabolic pathway and genotype-to-phenotype correlations are depicted in Fig 5. As shown in S7 Table, it is obvious that the colored phenotype of seeds (i.e., black seed coat/hilum and brown pod/pubescence) is exhibited when all these anthocyanins are synthesized, except for delphinidin-derived anthocyanins (e.g., delphinidin 3-O-gloucoside and petunidin 3-O-glucoside). The *W1* locus, in which the F3’5’H gene plays a key role, governs the production of delphinidin-derived anthocyanins and may affect colors of seeds and flowers. Thus, even if three other loci (i.e., *I*, *T* and *R*) located in the upstream of the metabolic pathway are all functionally active, the *w1* allele would prohibit the production of delphinidin-derived pigments, as represented in the phenotype of white flowers under the genetic background of *iiRRTTw1w1* allele combination (Fig 5). The *T* locus, which harbors the F3’H gene as the key enzyme, plays its role at the nodal position within the anthocyanin biosynthetic pathway, at which it can control the production of cyanidin-derived anthocyanins as well as delphinidin-derived ones (Fig 5). Consistent with this pathway-based depiction, 99.3% (146/147) soybeans with *t* allele exhibited yellow seed coats (S6B Table). In addition, the *R* locus (represented by R2R3 MYB TF) plays a key regulatory role before and after the synthesis of anthocyanidins (e.g., cyanidin and delphinidin) by modulating the expression of ANS and UF3GT. Finally, the *I* locus (represented by CHS gene clusters) occupies the very top hierarchy of the flavonoid pathway, and thus plays the key role as a master switch that can control overall anthocyanin biosynthesis. Therefore, if genes and/or alleles of high status within the biosynthetic pathway are in inactive forms (e.g., *i*^*i*^*i*^*i*^ or *tt* alleles), such genetic background will cause colorless phenotypes in seeds (Fig 5). However, since the phenotypes relevant to these multiple loci can be further confounded by other homologous genes residing in different genomic loci, integrative genetic dissection and in-depth interpretation should be made with a great caution.

As mentioned in the results section, rbGWAS could not capture the full content of association signals at the *I* locus due to incomplete assembly of the reference genome (Fig 2C). It was obvious that the defective structure of the current reference genome was caused by duplicated clusters of CHS genes around the *I* locus, but its structural arrangement of inverted repeats was demonstrated by previous analyses in soybean [12,57] and *G*. *soja* [58]. In both genomes, they share the same inversely repeated array composed of two I-clusters (i.e., I-cluster A; CHS1-3-4 and I-cluster B; CHS4-3-1) on the chromosome 8 [59]. As it is well known, CHS is one of the most important master switches that regulate the biosynthetic pathways for the anthocyanin production with the highest hierarchy (Fig 5). Thus, many endeavors have been made to get an insight into how the CHS cluster-containing *i*^*i*^ allele exerts its effect on seed colors via gene silencing. Both Tuteja et al (2009) and Xie et al (2019) had proposed their own working models for CHS gene silencing occurring at the *i*^*i*^ allele, but in different ways. Thus, it was interpreted with our extreme caution that both models did not seem fully explainable. Tuteja et al (2009) appeared to miss a fact that chimeric subtilisin-CHS1/CHS3 transcript was created and acted as a trigger for the PTGS. In contrast, Xie et al (2019) recognized the presence of the chimeric gene by comparing *G*. *soja* and *G*. *max* genomes. Nevertheless, it was suspected that they could not precisely define a new open reading frame for the chimeric transcript, by which could serve as an initiator for the inverted repeat-mediated PTGS.

**Fig 6:**
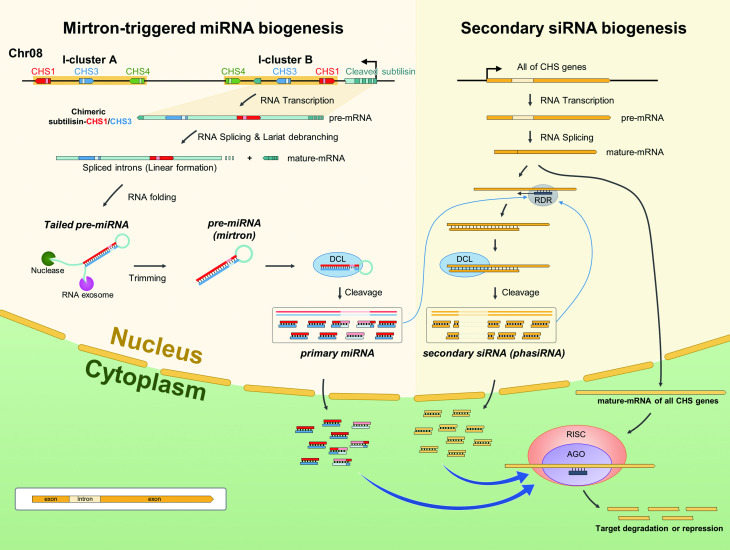
A proposed model for ‘Mirtron-Triggered Gene Silencing (MTGS)’ mechanism working on genome-wide CHS regulatory circuit. It is noteworthy that the mirtron-derived primary miRNAs involve intron segments originated from CHS1/CHS3-containing transcript whereas the secondary siRNAs are all generated from the exon regions of other CHS transcripts. Abbreviations are as follows: RISC, RNA-induced silencing complex; AGO, argonaute proteins; RDR, RNA dependent RNA polymerase; DCL, dicer-like protein.

By employing the k-mer analysis, this study independently revealed the recombination event occurred between subtilisin and I-cluster B and resulting structural change that had been inherited and adapted into the currently cultivated soybean genomes carrying the phenotype of yellow seed coat (Fig 3A). One step further, mapping analysis of mRNA-seq data allowed us to identify the chimeric SC1C3 transcript in the seed coat, whose pre-mRNA contained CHS1 and CHS3 within its newly acquired intron (Fig 3B). Subsequently, small RNA mapping analysis against CHS genes revealed the generation of miRNAs from the intron composed of CHS1 and CHS3, but not from introns of CHS4 and other CHS genes (Fig 4, S7 Fig), and hence providing a significant evidence for the mirtron production. Such a special type of miRNA precursor (i.e., mirtron) has recently been found and reported in many species, such as *Drosophila*, *C*. *elegans*, Human, Arabidopsis and Rice [48,60–62]. Taken together with these two new findings and the special structural feature of the *i*^*i*^ allele, we are carefully proposing a new PTGS working model via ‘mirtron-triggered CHS gene silencing (MTGS)’ mechanism, which would be occurring in the yellow seed soybeans (Fig 6). As demonstrated in Fig 6, the chimeric SC1C3 gene generates its pre-mRNA containing a new transcript frame under the control of subtilisin promoter, thereafter producing a lariat form of post-splicing introns, one of which harbors CHS1 and CHS3 arranged in an inverted orientation. After the lariat-form intron is linearized by a debranching enzyme (i.e., debranchase), the CHS1/CHS3-containing intron will fold into stem/loop-containing precursors (tailed pre-miRNA) due to its innate structural feature of inverted tandem duplication. Subsequently, 3’ and 5’ overhangs of tailed pre-miRNAs are removed by RNA exosome and nucleases, respectively [60,63–66], finally generating hairpin-formed intron-containing miRNA precursors (i.e., mirtron) [48]. According to known general PTGS process in plants, the double-stranded stem portion of mirtron is digested into short miRNA by Dicer-like protein (DCL), which occurs in the nucleus [67]. Furthermore, based on CHS-wide small RNA mapping analysis (S7 Fig), it is strongly assumed that the primary miRNAs can serve as primers for the induction and production of phased, secondary, small interfering RNAs (i.e., phasiRNAs), which were originally designated as trans-acting small RNAs (tasiRNAs) and known as an efficient mechanism for synchronously controlling a large number of genes with some extents of homology (e.g., family genes such as MYB and NBS-LRR) [49,68]. Similar to this mechanism, phasiRNA (or secondary siRNA) derived from other CHS genes can be generated in such a way that the single-stranded primary miRNA act as primers for the RNA-dependent RNA polymerase (RDR), and subsequently resulting double-stranded RNA precursors are diced by the DCL proteins, finally producing the secondary siRNA (i.e., phasiRNA) (Fig 6).

In this way, it appears that the pool of small RNA species (i.e., primary miRNA plus secondary phasiRNA) can be enormously amplified through these two separate, but actually interacting, transcription regulatory circuits (Fig 6), until the cell is equipped with nearly the entire breadth of CHS gene-wide sequence specificity, which was evidenced by the small RNA mapping analyses (S7 Fig). A possible scenario is that such a pool of small RNAs with full extent of CHS-wide specificity may be able to degrade almost all of CHS transcripts expressed from all genome-wide sectors for the CHS genes, ultimately leading to the phenotype (i.e., colorlessness) caused by complete knock-down of CHS genes. Consistent with this scenario, we could not detect any anthocyanins in yellow soybeans (Fig 5, S7 Table). In this working model, it is noteworthy that the mirtron plays a critical role as a ‘trigger’ for the PTGS-mediated regulatory circuit (Fig 6).

## Conclusions

To precisely identify and dissect genes and genomic loci associated with the pigmentation of soybean seeds, this study employed an integrated approach in both analytical means (i.e., rbGWAS and rfGWAS) and data resources (i.e., in-house-generated and public WGR data). These analyses resulted in the detection of four major genomic loci (designated as *SP1* ~ *SP4*), key genes and numerous functional variants across the soybean whole genome. Of these four loci, in-depth dissection of *SP3* locus (conventionally known as *I* locus), in which contained a special genomic arrangement of CHS gene clusters, led us to a meaningful finding. The k-mer analysis allowed us to discover an ancient recombination event between subtilisin and I-cluster B, leading to the revelation of special structural features in the *i*^*i*^ allele of *I* locus. Mapping analyses with NGS-derived mRNA-seq and small RNA data enabled us to discover chimeric subtilisin-CHS1/CHS3 transcripts followed by ‘mirtron’ generation. Based on these results, it implicates that the mirtron plays a pivotal role as an initiator towards the direction of amplifying CHS-derived secondary siRNAs or phasiRNA, thereafter leading to the complete silencing of genome-wide CHS genes. Consequently, we carefully suggest a new working model of ‘mirtron-triggered gene silencing’ (or MTGS) mechanism, even if it needs to be further investigated through future experimental endeavor. It is anticipated that the MTGS model will offer a deeper and broader insight into the PTGS pathways interacting within the context of complex regulatory circuits. We also expect that the information on trait-associated alleles found in this study will be beneficially used for the precision breeding of the crop soybean.

## Supporting information

S1 Fig The population structure of 438 soybean accessions. The fastSTRUCTURE program was used to infer the structure of all Glycine accessions used in this study. Each group is presented by different colors.
(PPTX)

S2 Fig Genome-wide distribution of genes and variations. Both gene densities and frequencies of SNP/InDel were all depicted by counting its corresponding number of genes and variants every 100Kbp-long genomic section.
(PPTX)

S3 Fig GWAS analyses and Manhattan plots resulted from all possible combinations of the seed color-associated traits. (PPTX)

S4 Fig GWAS analyses and Manhattan plots associated with pod color, follower color and growth habit. (PPTX)

S5 Fig GWAS analysis of SP1 locus and structural comparisons of CaaXEP gene and protein. (A) Comparison of the green seed coat-linked GWAS analyses and consistency of the Manhattan plots between the WGR data (248 soybean accessions) and SoySNP50K data (10312 soybean accessions). Color-coded circles indicate the functional impacts of variants, as denoted in Fig 2. In the magnified image, Glyma.01G198500 (CaaX-type endopeptidase; CaaXEP) was expected to be a candidate with the most functionally significant variation and thereby further analyzed. (B) Comparison of transcriptome-based gene models for the CaaXEP. Each CaaXEP gene model for the yellow (upper) or green (below) soybean was depicted along with the RNA-seq read depths. (C) Modeling-based prediction of the 3D-structures and comparison of the CaaXEP proteins.
(PPTX)

S6 Fig Schematic of the genetic interactions between I locus and T locus, and expected phenotypes. Each color denotes actual phenotypes in the corresponding tissues, except for the white, which represents independent inheritance in corresponding part of the tissues.
(PPTX)

S7 Fig Comparison of small RNA mapping analyses within the phylogenetic context of nine representative CHS genes. The gray ribbons denote the genomic regions of perfect matches longer than 20 bps between the neighboring CHS genes. The gene sizes and positions of exon/intron junctions are all drawn to scale, while the read depth to the logarithmic scale.
(PPTX)

S1 Table List of phenotypic and NGS data information for 438 soybean accessions. (XLSX)

S2 Table Summary of genome-wide SNP/InDel distributions. (XLSX)

S3 Table Phenotypic distribution among three phylogenetic groups. (XLSX)

S4 Table List of variants identified at SP1 locus. (XLSX)

S5 Table Information for six candidate genes discovered at SP1 locus. (XLSX)

S6 Table Data consistency of genotypes between mapping-based and Kmer-based analyses. (XLSX)

S7 Table Phenotypes and anthocyanin quantification in seeds of 43 soybean accessions. (XLSX)
