# Supplementary material for: Dissecting seed pigmentation-associated genomic loci and genes by employing dual approaches of reference-based and k-mer-based GWAS with 438 Glycine accessions
Source: PLoS One. 2020 Dec 1;15(12):e0243085. doi: 10.1371/journal.pone.0243085 (PMC7707508; doi:10.1371/journal.pone.0243085)
Supplement: S1 Fig — The fastSTRUCTURE program was used to infer the structure of all Glycine accessions used in this study. Each group is presented by different colors. (PPTX) [file pone.0243085.s001.pptx]

## Slide 1
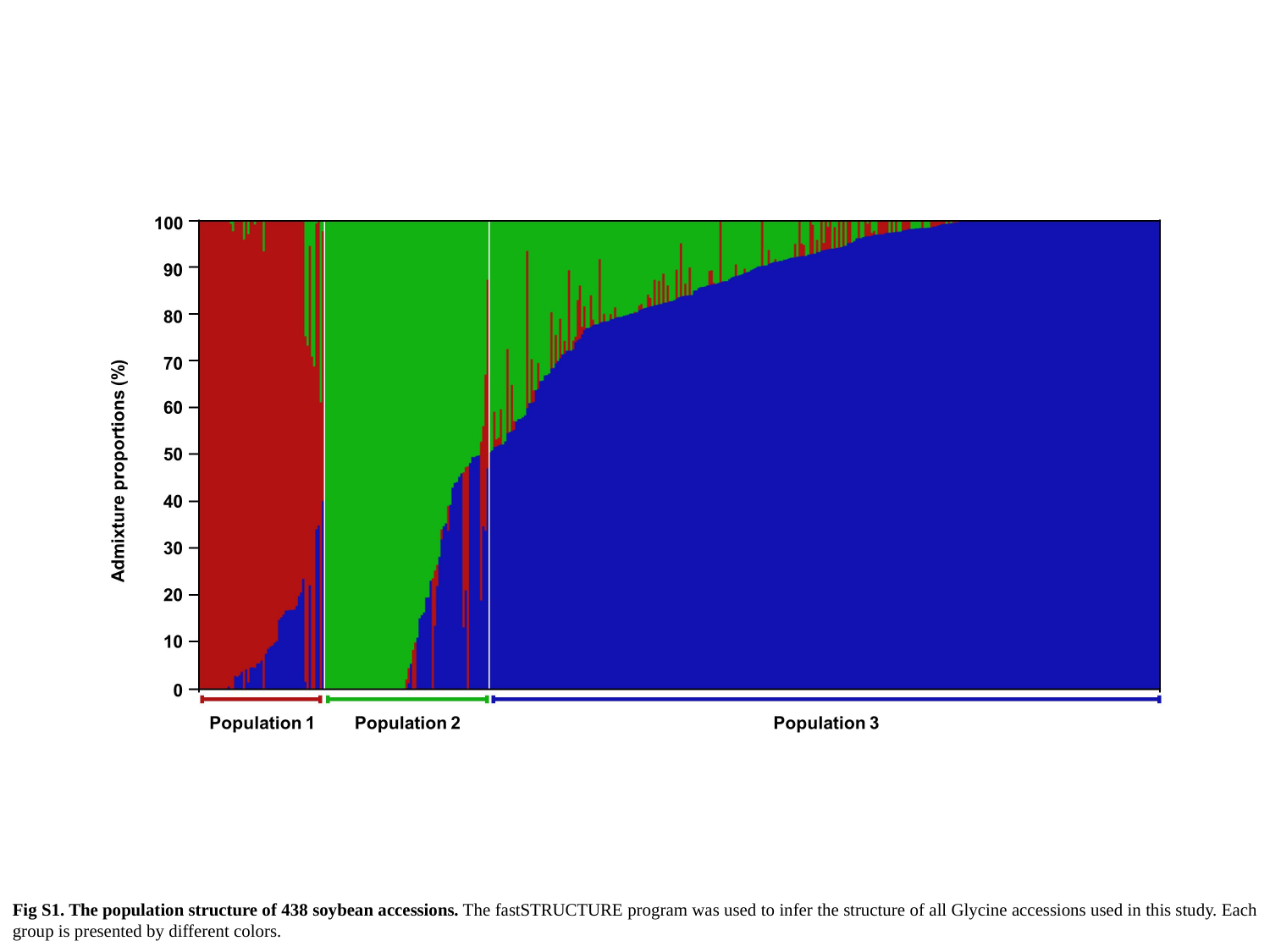

Fig S1. The population structure of 438 soybean accessions. The fastSTRUCTURE program was used to infer the structure of all Glycine accessions used in this study. Each group is presented by different colors.
