# Supplementary material for: Dissecting seed pigmentation-associated genomic loci and genes by employing dual approaches of reference-based and k-mer-based GWAS with 438 Glycine accessions
Source: PLoS One. 2020 Dec 1;15(12):e0243085. doi: 10.1371/journal.pone.0243085 (PMC7707508; doi:10.1371/journal.pone.0243085)
Supplement: S2 Fig — Both gene densities and frequencies of SNP/InDel were all depicted by counting its corresponding number of genes and variants every 100Kbp-long genomic section. (PPTX) [file pone.0243085.s002.pptx]

## Slide 1
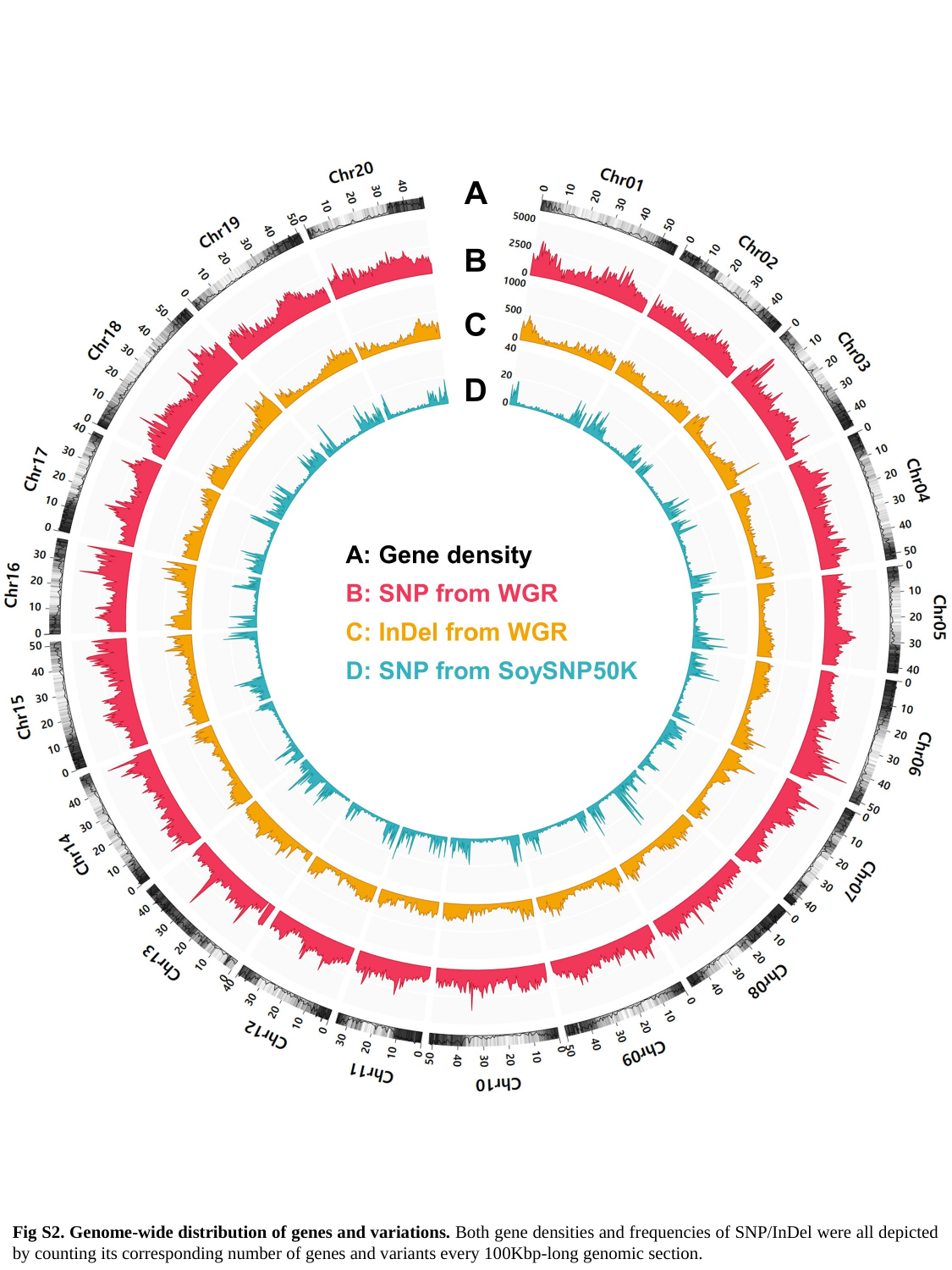

Fig S2. Genome-wide distribution of genes and variations. Both gene densities and frequencies of SNP/InDel were all depicted by counting its corresponding number of genes and variants every 100Kbp-long genomic section.
