# Supplementary material for: Dissecting seed pigmentation-associated genomic loci and genes by employing dual approaches of reference-based and k-mer-based GWAS with 438 Glycine accessions
Source: PLoS One. 2020 Dec 1;15(12):e0243085. doi: 10.1371/journal.pone.0243085 (PMC7707508; doi:10.1371/journal.pone.0243085)
Supplement: S3 Fig — (PPTX) [file pone.0243085.s003.pptx]

## Slide 1
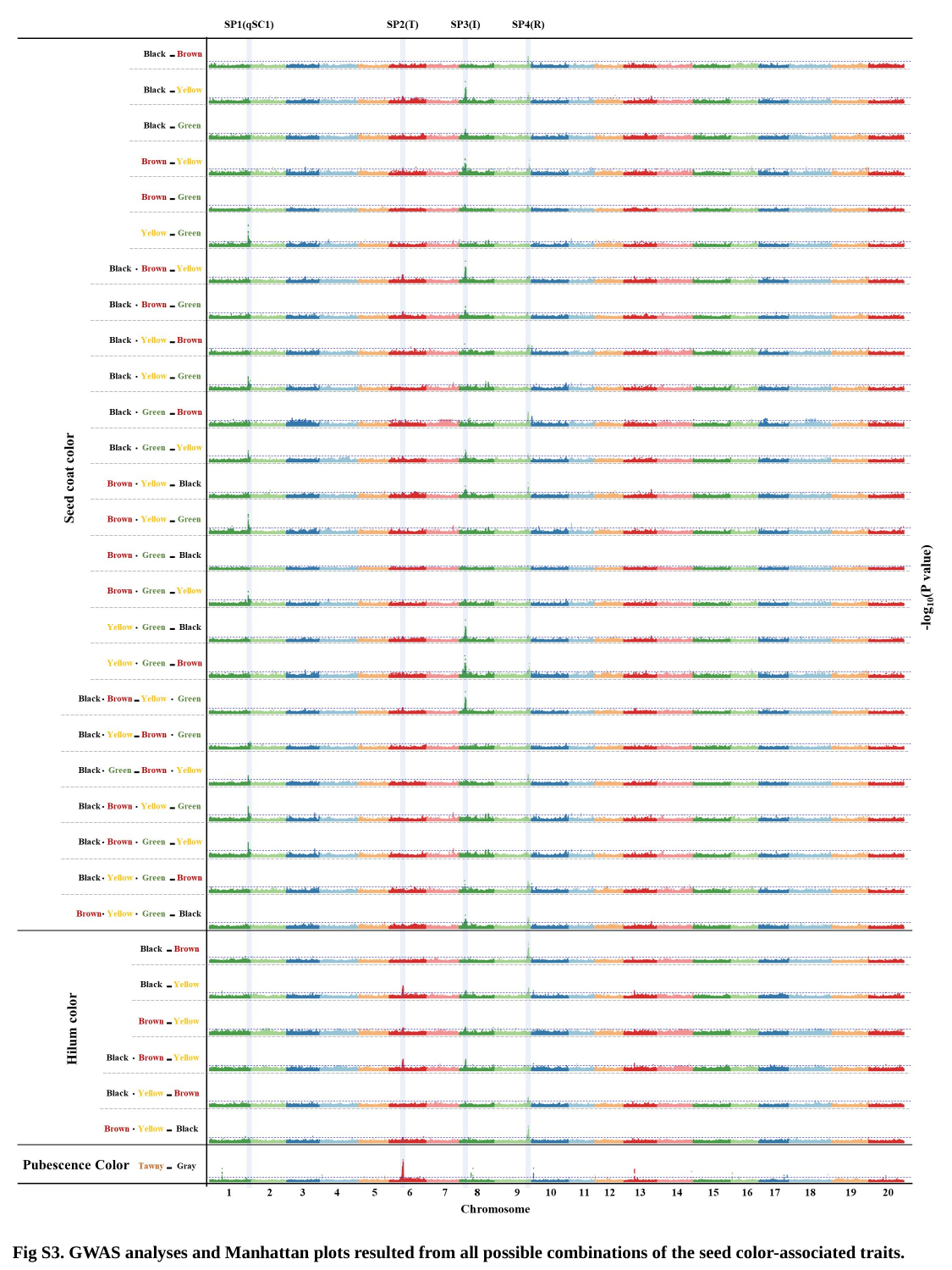

Fig S3. GWAS analyses and Manhattan plots resulted from all possible combinations of the seed color-associated traits.
