# Supplementary material for: Dissecting seed pigmentation-associated genomic loci and genes by employing dual approaches of reference-based and k-mer-based GWAS with 438 Glycine accessions
Source: PLoS One. 2020 Dec 1;15(12):e0243085. doi: 10.1371/journal.pone.0243085 (PMC7707508; doi:10.1371/journal.pone.0243085)
Supplement: S4 Fig — (PPTX) [file pone.0243085.s004.pptx]

## Slide 1
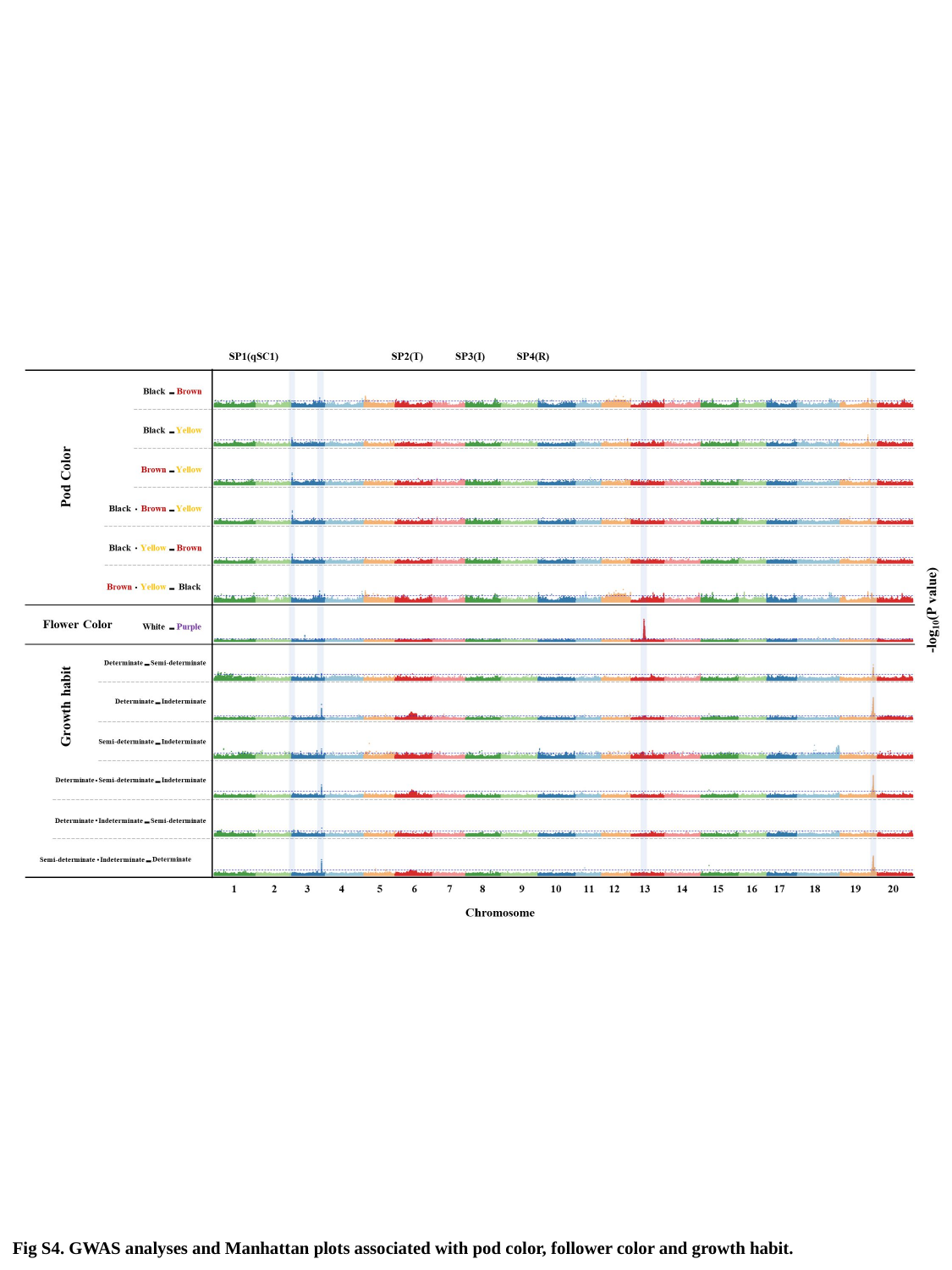

Fig S4. GWAS analyses and Manhattan plots associated with pod color, follower color and growth habit.
