# Supplementary material for: Dissecting seed pigmentation-associated genomic loci and genes by employing dual approaches of reference-based and k-mer-based GWAS with 438 Glycine accessions
Source: PLoS One. 2020 Dec 1;15(12):e0243085. doi: 10.1371/journal.pone.0243085 (PMC7707508; doi:10.1371/journal.pone.0243085)
Supplement: S5 Fig — (A) Comparison of the green seed coat-linked GWAS analyses and consistency of the Manhattan plots between the WGR data (248 soybean accessions) and SoySNP50K data (10312 soybean accessions). Color-coded circles indicate the functional impacts of variants, as denoted in Fig 2. In the magnified image, Glyma.01G198500 (CaaX-type endopeptidase; CaaXEP) was expected to be a candidate with the most functionally significant variation and thereby further analyzed. (B) Comparison of transcriptome-based gene models for the CaaXEP. Each CaaXEP gene model for the yellow (upper) or green (below) soybean was depicted along with the RNA-seq read depths. (C) Modeling-based prediction of the 3D-structures and comparison of the CaaXEP proteins. (PPTX) [file pone.0243085.s005.pptx]

## Slide 1
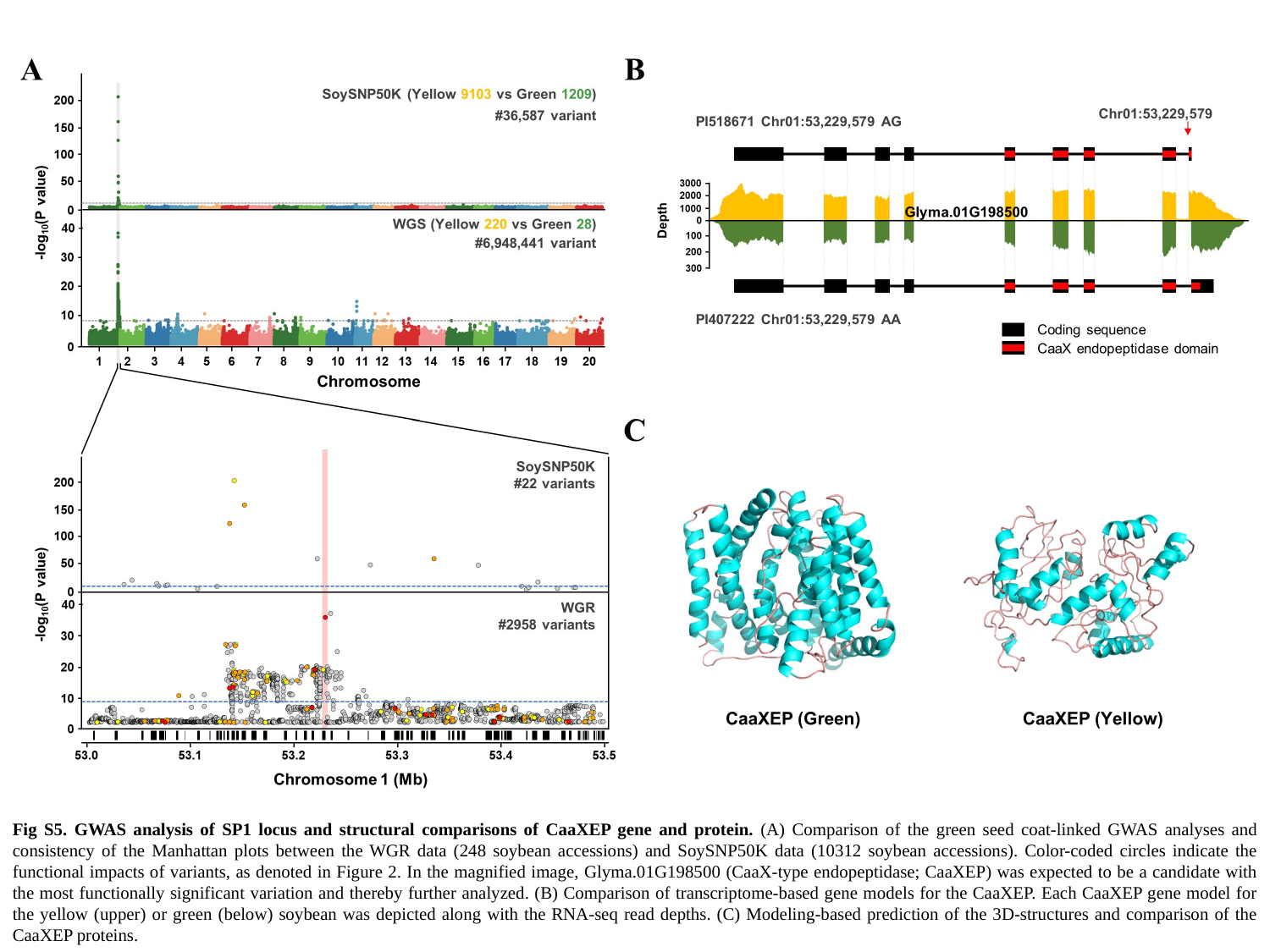

Fig S5. GWAS analysis of SP1 locus and structural comparisons of CaaXEP gene and protein. (A) Comparison of the green seed coat-linked GWAS analyses and consistency of the Manhattan plots between the WGR data (248 soybean accessions) and SoySNP50K data (10312 soybean accessions). Color-coded circles indicate the functional impacts of variants, as denoted in Figure 2. In the magnified image, Glyma.01G198500 (CaaX-type endopeptidase; CaaXEP) was expected to be a candidate with the most functionally significant variation and thereby further analyzed. (B) Comparison of transcriptome-based gene models for the CaaXEP. Each CaaXEP gene model for the yellow (upper) or green (below) soybean was depicted along with the RNA-seq read depths. (C) Modeling-based prediction of the 3D-structures and comparison of the CaaXEP proteins.
