# Supplementary material for: Dissecting seed pigmentation-associated genomic loci and genes by employing dual approaches of reference-based and k-mer-based GWAS with 438 Glycine accessions
Source: PLoS One. 2020 Dec 1;15(12):e0243085. doi: 10.1371/journal.pone.0243085 (PMC7707508; doi:10.1371/journal.pone.0243085)
Supplement: S6 Fig — Each color denotes actual phenotypes in the corresponding tissues, except for the white, which represents independent inheritance in corresponding part of the tissues. (PPTX) [file pone.0243085.s006.pptx]

## Slide 1
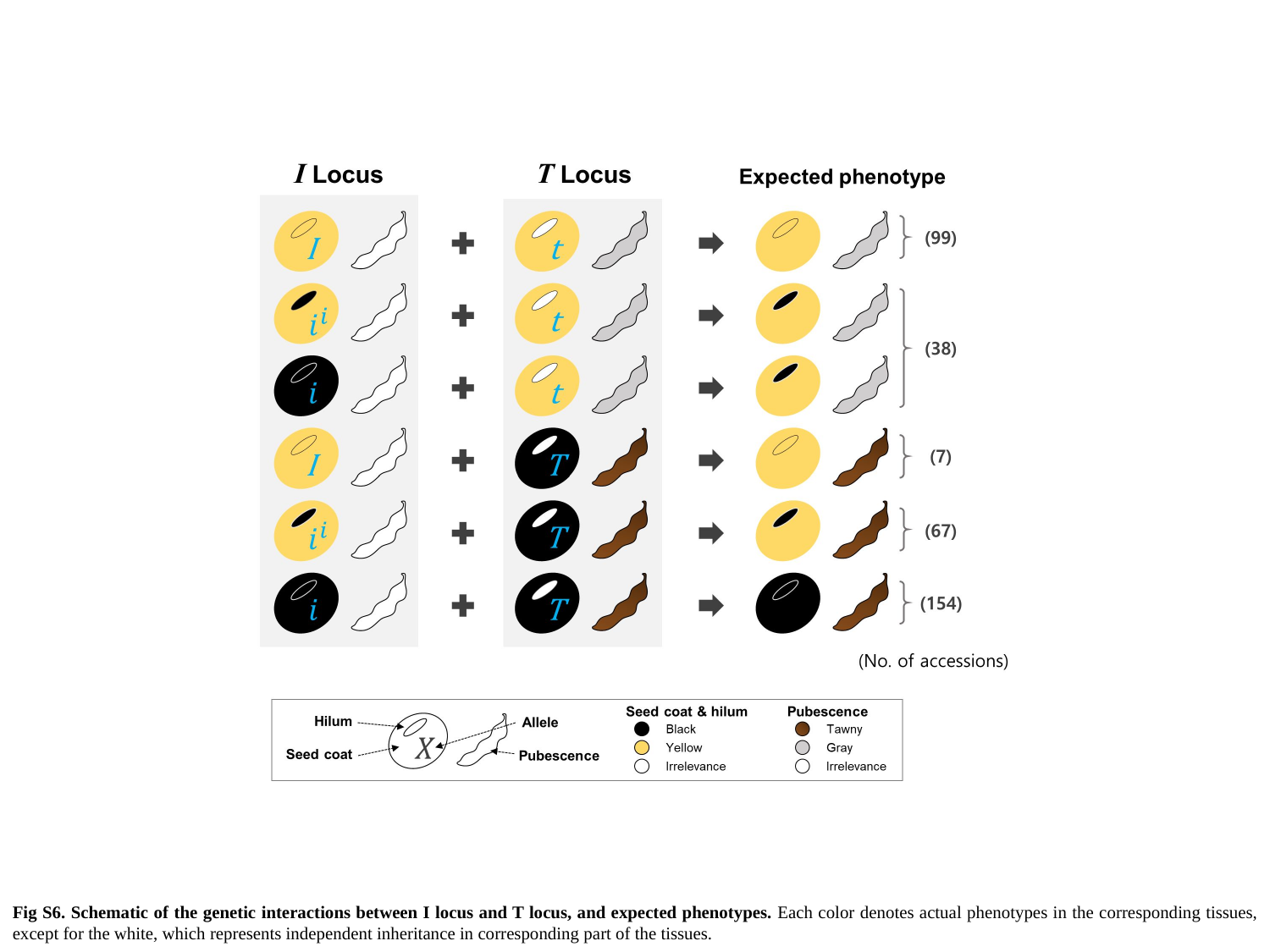

Fig S6. Schematic of the genetic interactions between I locus and T locus, and expected phenotypes. Each color denotes actual phenotypes in the corresponding tissues, except for the white, which represents independent inheritance in corresponding part of the tissues.
