# Supplementary material for: Dissecting seed pigmentation-associated genomic loci and genes by employing dual approaches of reference-based and k-mer-based GWAS with 438 Glycine accessions
Source: PLoS One. 2020 Dec 1;15(12):e0243085. doi: 10.1371/journal.pone.0243085 (PMC7707508; doi:10.1371/journal.pone.0243085)
Supplement: S7 Fig — The gray ribbons denote the genomic regions of perfect matches longer than 20 bps between the neighboring CHS genes. The gene sizes and positions of exon/intron junctions are all drawn to scale, while the read depth to the logarithmic scale. (PPTX) [file pone.0243085.s007.pptx]

## Slide 1
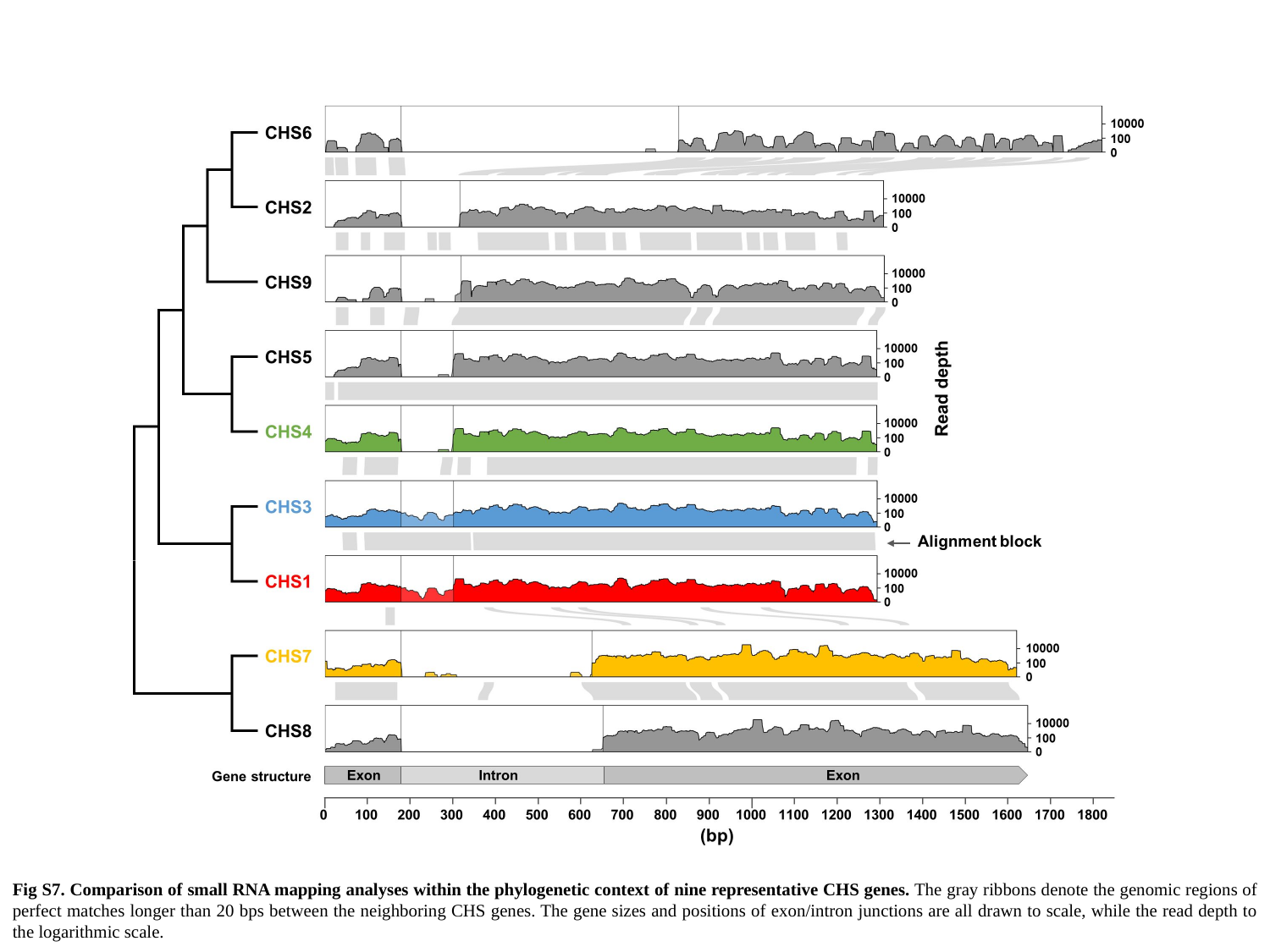

Fig S7. Comparison of small RNA mapping analyses within the phylogenetic context of nine representative CHS genes. The gray ribbons denote the genomic regions of perfect matches longer than 20 bps between the neighboring CHS genes. The gene sizes and positions of exon/intron junctions are all drawn to scale, while the read depth to the logarithmic scale.
